# Supplementary material for: Manipulating the oxygen reduction reaction pathway on Pt-coordinated motifs
Source: Nat Commun. 2022 Feb 3;13:685. doi: 10.1038/s41467-022-28346-0 (PMC8813992; doi:10.1038/s41467-022-28346-0)
Supplement: Supplementary file 1 — Supplementary Information [file 41467_2022_28346_MOESM1_ESM.docx]

Supplementary Information for

Manipulating the oxygen reduction reaction pathway on Pt-coordinated motifs

Jiajun Zhao,^1,2^ Cehuang Fu,^1^ Ke Ye,^1,2^ Zheng Liang,^3^ Fangling Jiang,^4^ Shuiyun Shen,^1^ Xiaoran Zhao,^5^ Lu Ma,^6^ Zulipiya Shadike,^1^ Xiaoming Wang,^7^ Junliang Zhang,^1,^* Kun Jiang^1,2^*

^1^Institute of Fuel Cells, School of Mechanical Engineering, Shanghai Jiao Tong University, Shanghai 200240, China.

^2^Interdisciplinary Research Center, School of Mechanical Engineering, Shanghai Jiao Tong University, Shanghai 200240, China.

^3^Laboratory of Energy Chemical Engineering, Frontiers Science Center for Transformative Molecules, School of Chemistry and Chemical Engineering, Shanghai Jiao Tong University, Shanghai, 200240, China.

^4^State Key Laboratory of High-Performance Ceramics and Superfine Microstructure, Shanghai Institute of Ceramics, Chinese Academy of Sciences, Shanghai 201899, China.

^5^Shanghai Key Laboratory of Advanced High-Temperature Materials and Precision Forming, State Key Laboratory of Metal Matrix Composites, School of Materials Science and Engineering, Shanghai Jiao Tong University, Shanghai 200240, China.

^6^National Synchrotron Light Source II, Brookhaven National Laboratory, Upton, NY11973, USA.

^7^Department of Chemistry and Key Laboratory for Preparation and Application of Ordered Structural Materials of Guangdong Province, Shantou University, Shantou 515063, China.

^*^ To whom correspondence should be addressed: J.Z. (Email: [junliang.zhang@sjtu.edu.cn](mailto:junliang.zhang@sjtu.edu.cn)) or K.J. (Email: [kunjiang@sjtu.edu.cn](mailto:kunjiang@sjtu.edu.cn))

**Supplementary Figures**


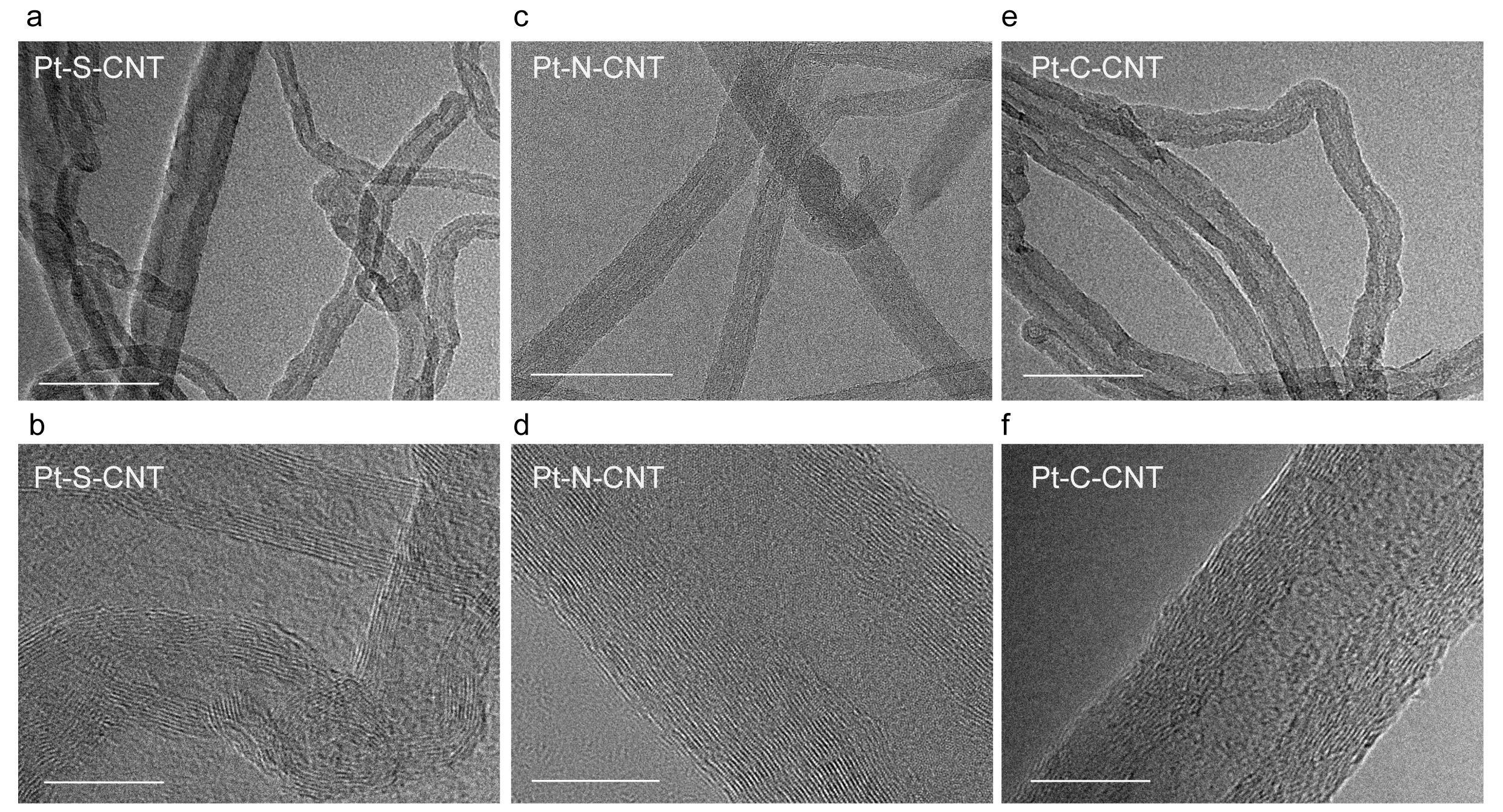


**Supplementary Figure 1.** Bright field TEM images of (**a**-**b**) Pt-S-CNT, (**c**-**d**) Pt-N-CNT, and (**e**-**f**) Pt-C-CNT. Scale bars for (**a**, **c**, **e**), 50 nm, for (**b**, **d**, **f**), 10 nm. No metal clusters or nanoparticles were observed.


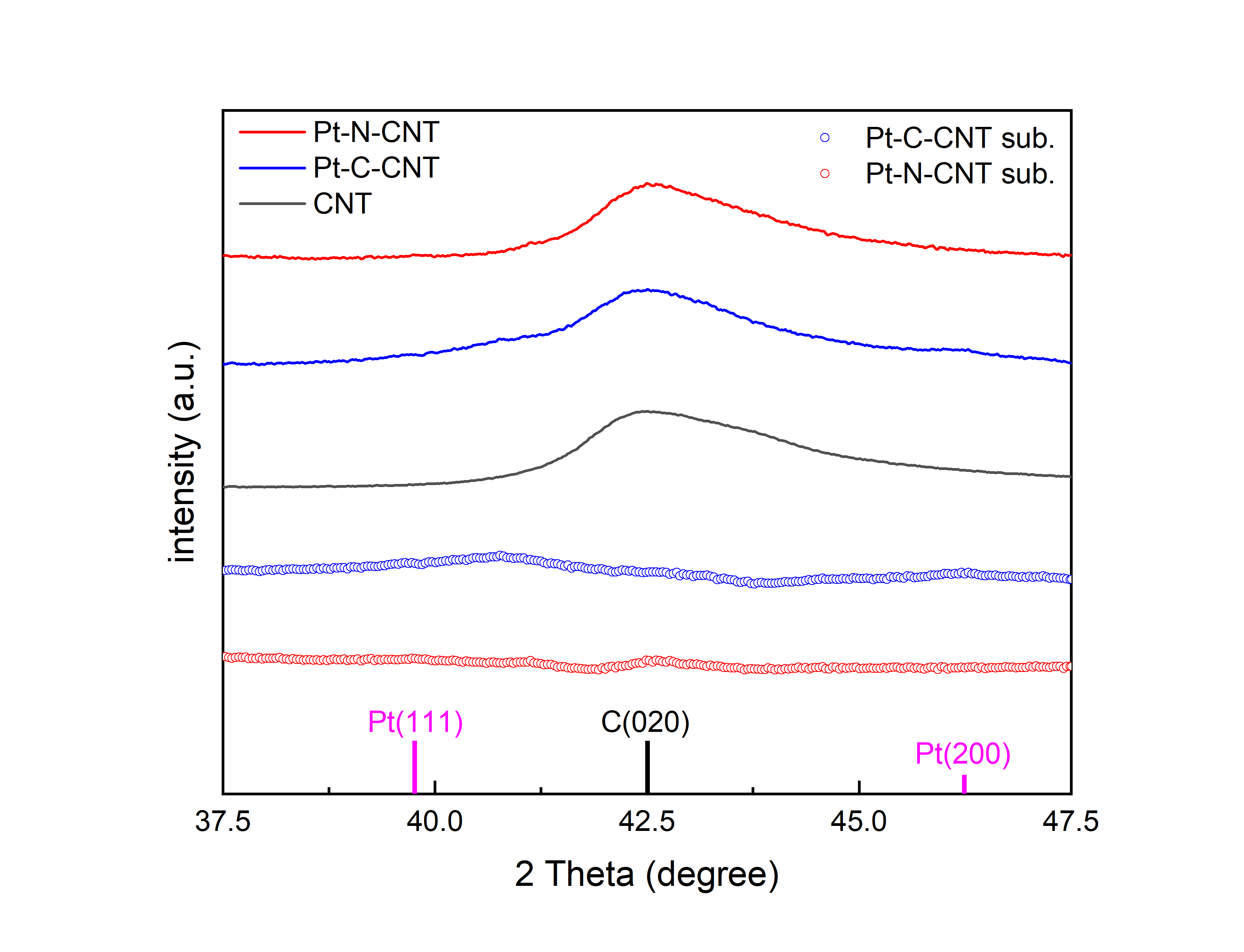


**Supplementary Figure 2**. High resolution X-ray diffraction (XRD) patterns of Pt-N-CNT, Pt-C-CNT and CNT. To better illustrate if any Pt diffraction feature was embedded in the broad peak of carbon, we further performed the spectra subtraction using bare CNT as reference, and the results were shown as dotted lines. The insert histograms refer to standard Pt diffraction of PDF No. 00-004-0802 and C diffraction of PDF No. 00-054-0501.

**
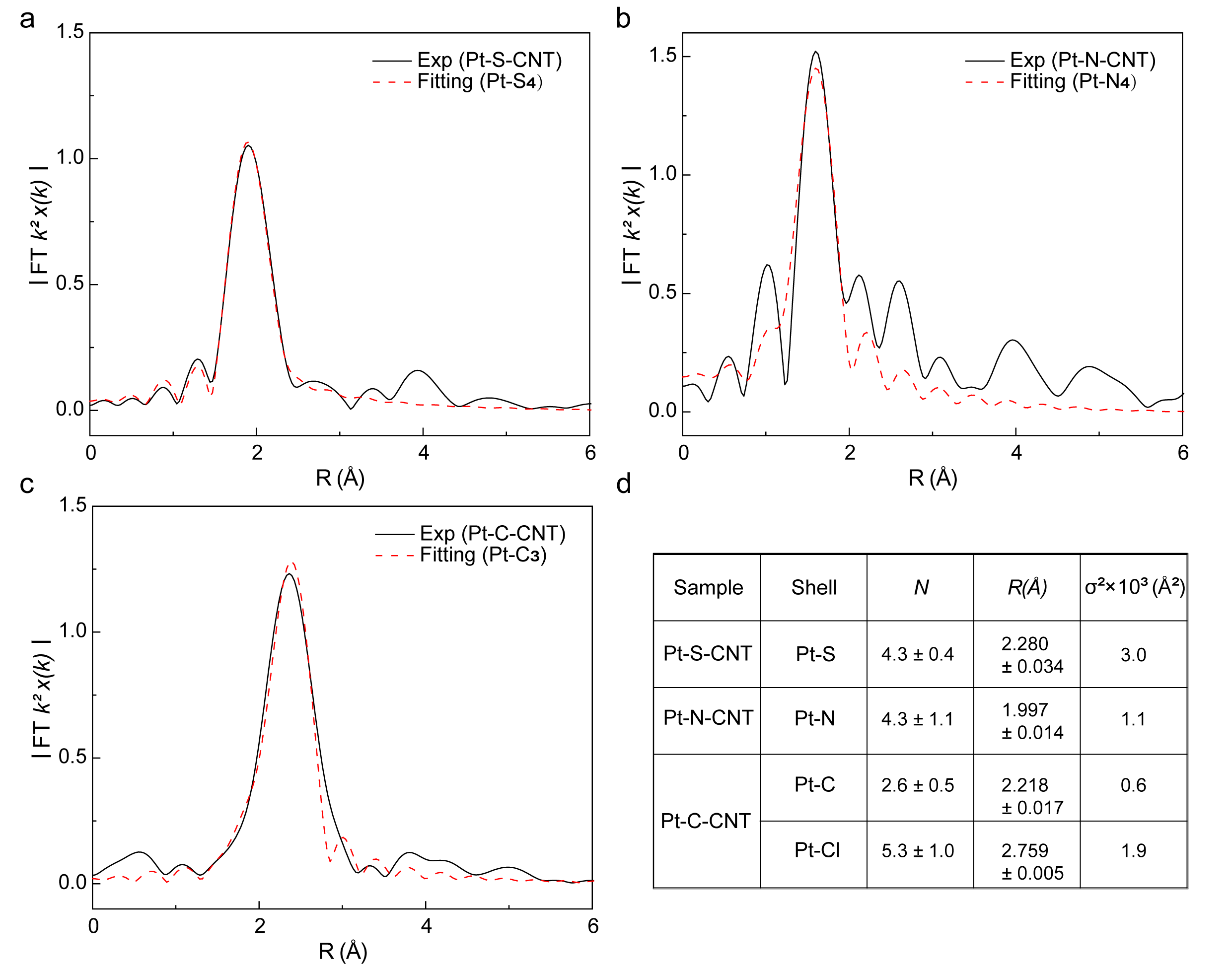
**

**Supplementary Figure 3.** FT-EXAFS fitting curves of Pt-X-CNT at the Pt L_3_-edge using (**a**) Pt-S path, (**b**) Pt-N path, (**c**) mixed Pt-C and Pt-Cl paths, together with (**d**) the EXAFS fitting parameters. For the Pt-C-CNT catalyst, a coordination dopant larger than C/N/O is expected to contribute to the observed peak at ~2.3 Å, possibly arisen from the residual chloride species from the K_2_PtCl_4_ precursor.

**
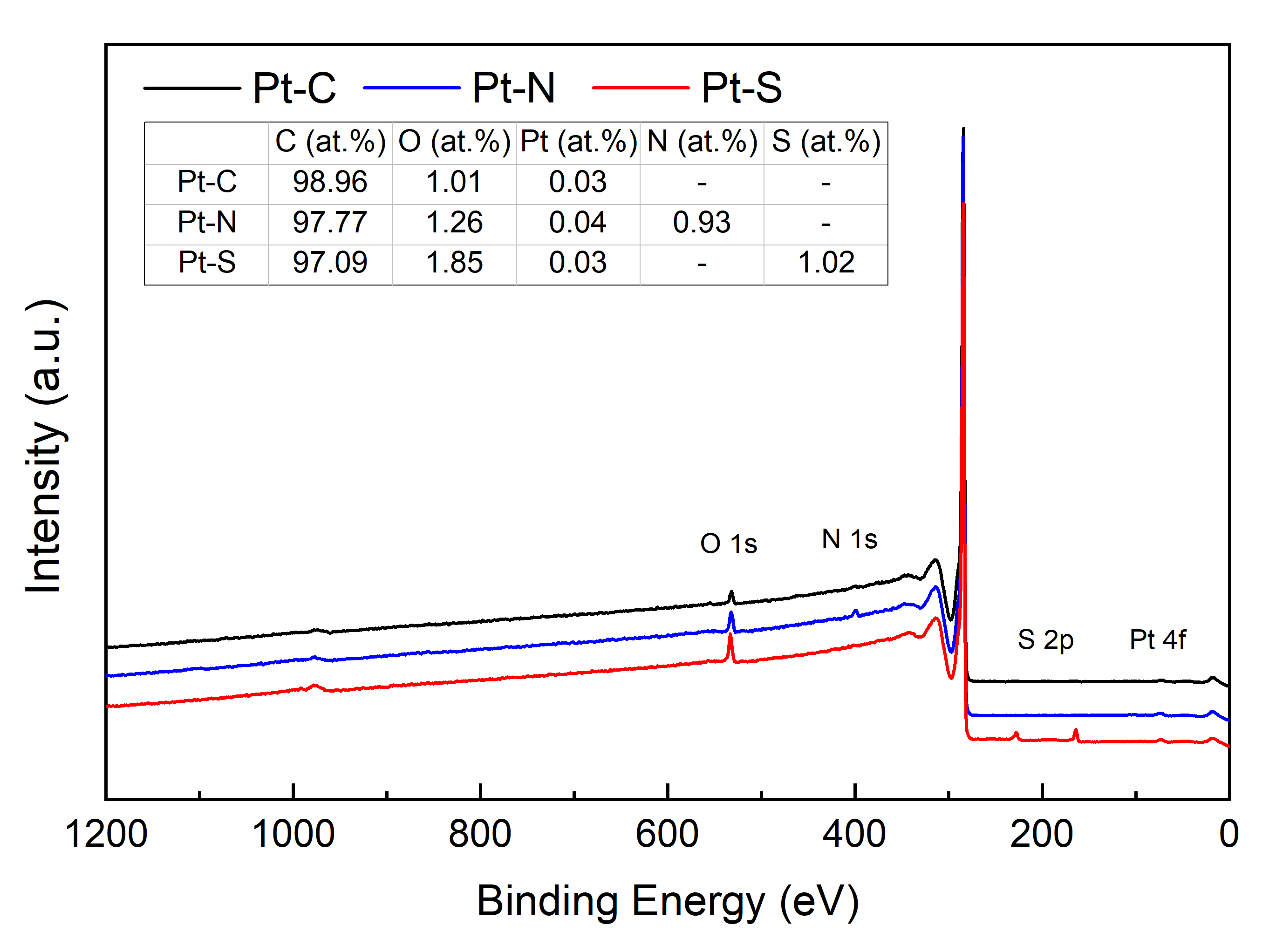
**

**Supplementary Figure 4.** XPS survey spectra for Pt-X-CNT catalysts with different coordination elements. C, O and Pt elements were detected in all samples, while N component was detected at ~400 eV in Pt-N-CNT and S at ~228 and ~165 eV in Pt-S-CNT. The atomic content of each element is tabulated as insert.


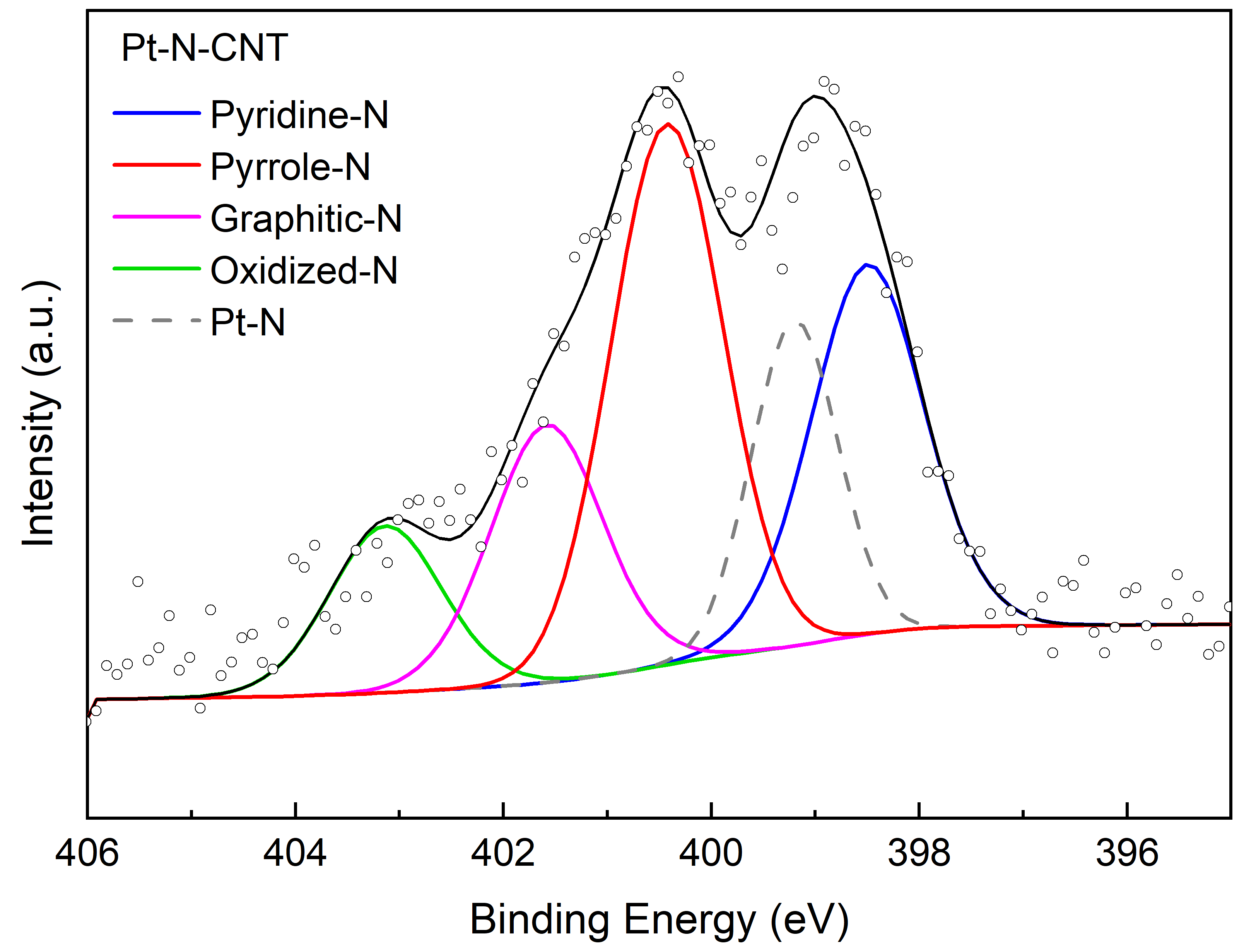


**Supplementary Figure 5.** Core level XPS on N 1s region for Pt-N-CNT. Five different N-coordination motifs could be deconvoluted: pyridine-N (398.5 eV, 23.55%), pyrrole-N (400.4 eV, 33.81%), graphitic-N (401.6 eV, 16.40%) oxidized-N (403.1 eV, 10.63%) and Pt-N (399.2 eV,15.62%)^1,2^.


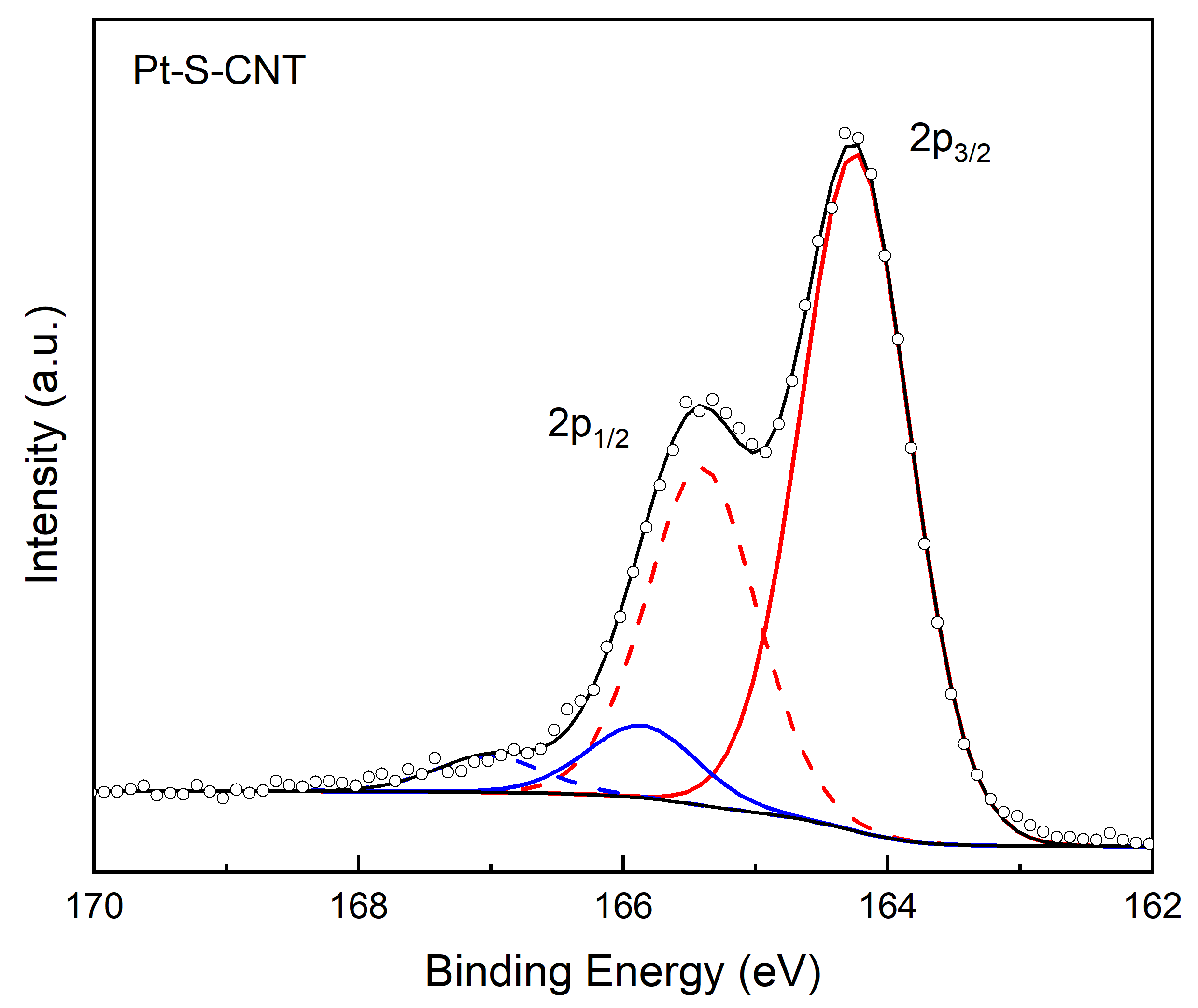


**Supplementary Figure 6.** Core level XPS on S 2p region for 200-Pt-S-CNT. The peak positioned at 164.2 eV can be associated with sulfur directly bonded to carbon atoms in the form of C−S−C, and the peak observed at 165.9 eV can be associated with SO_x_ species bonded to carbon^3^.


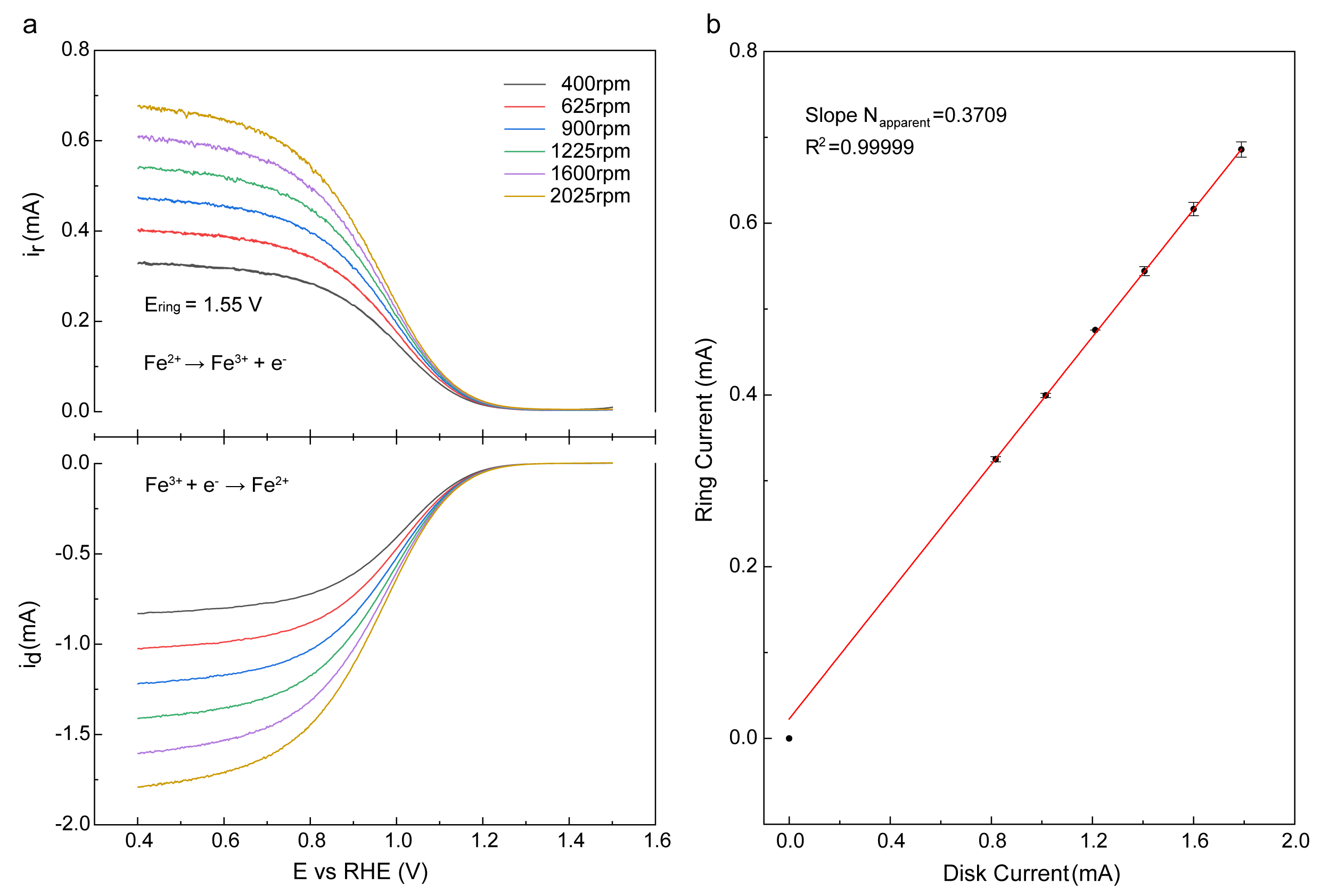


**Supplementary Figure 7.** RRDE collection efficiency calibration. (**a**) Linear sweep voltammetric curves recorded on a bare glassy carbon rotation disk electrode (*Φ* = 5.61 mm) with a Pt ring (*Φ* = 7.91 mm) in the electrolyte of 0.1 M KOH + 10 mM K_3_Fe(CN)_6_ . Sweep rate: 20 mV s^−1^, *E*_ring_ = 1.55 V vs RHE. (**b**) Linear fitting of the diffusion limited current densities recorded on ring and disk electrodes at different rotation speed. The experimental determined apparent collection efficiency (*N*) is 37.09%, close to the theoretical vale of 37.00% (Pine AFE7R9).


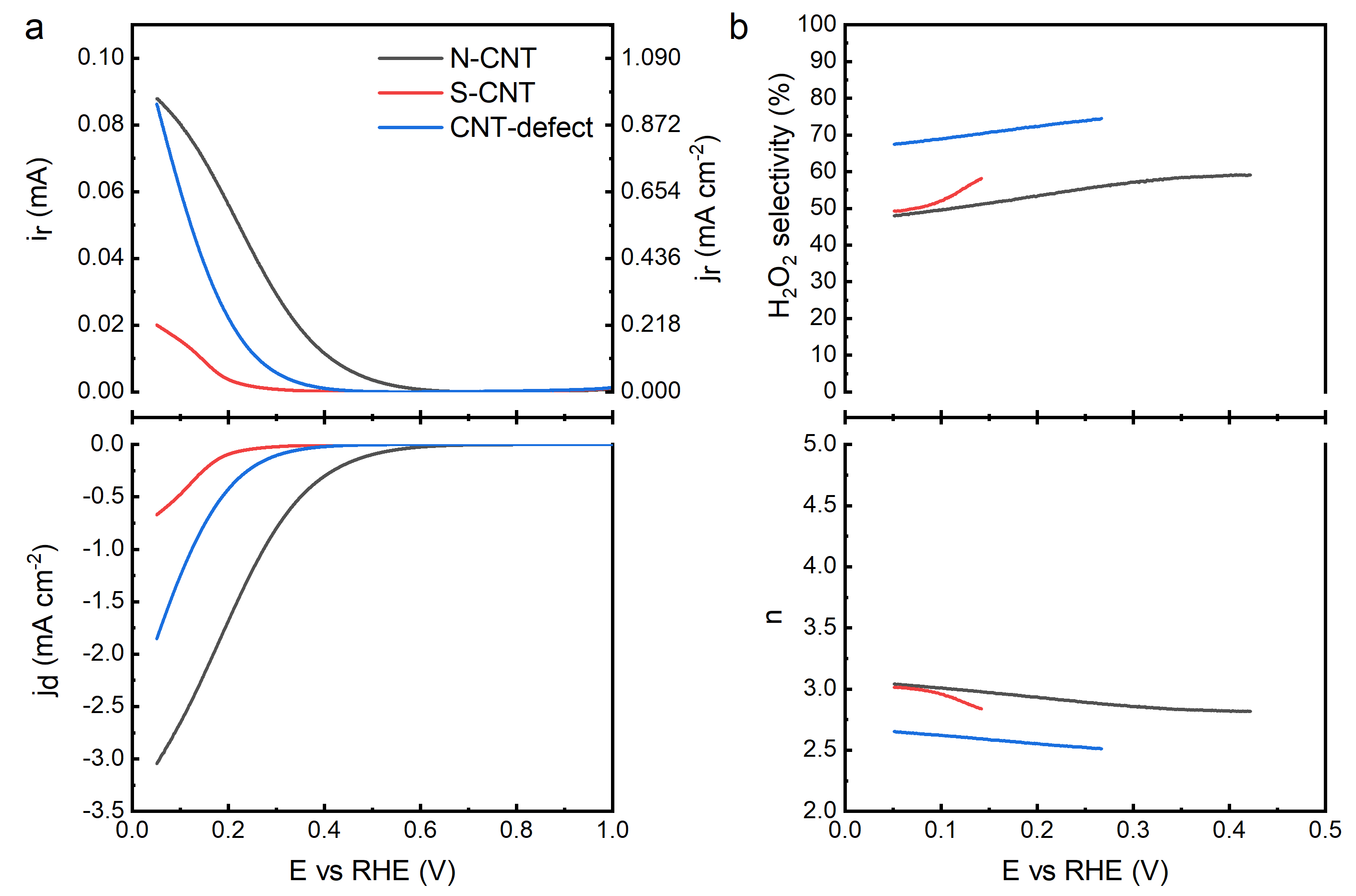


**Supplementary Figure 8.** ORR performance of X-CNT doped with different metalloid component. (**a**) LSVs of N-CNT, S-CNT and defective CNT recorded at 1600 rpm and a scan rate of 5 mV s^−1^, together with the detected H_2_O_2_ currents on the ring electrode (upper panel) at a fixed potential of 1.2 V vs. RHE. (**b**) Calculated H_2_O_2_ selectivity and electron transfer number during negative going potential sweep. H_2_O_2_ selectivity and *n* were plotted from the onset potential that reaching 0.1 mA cm^-2^ H_2_O_2_ partial current density.


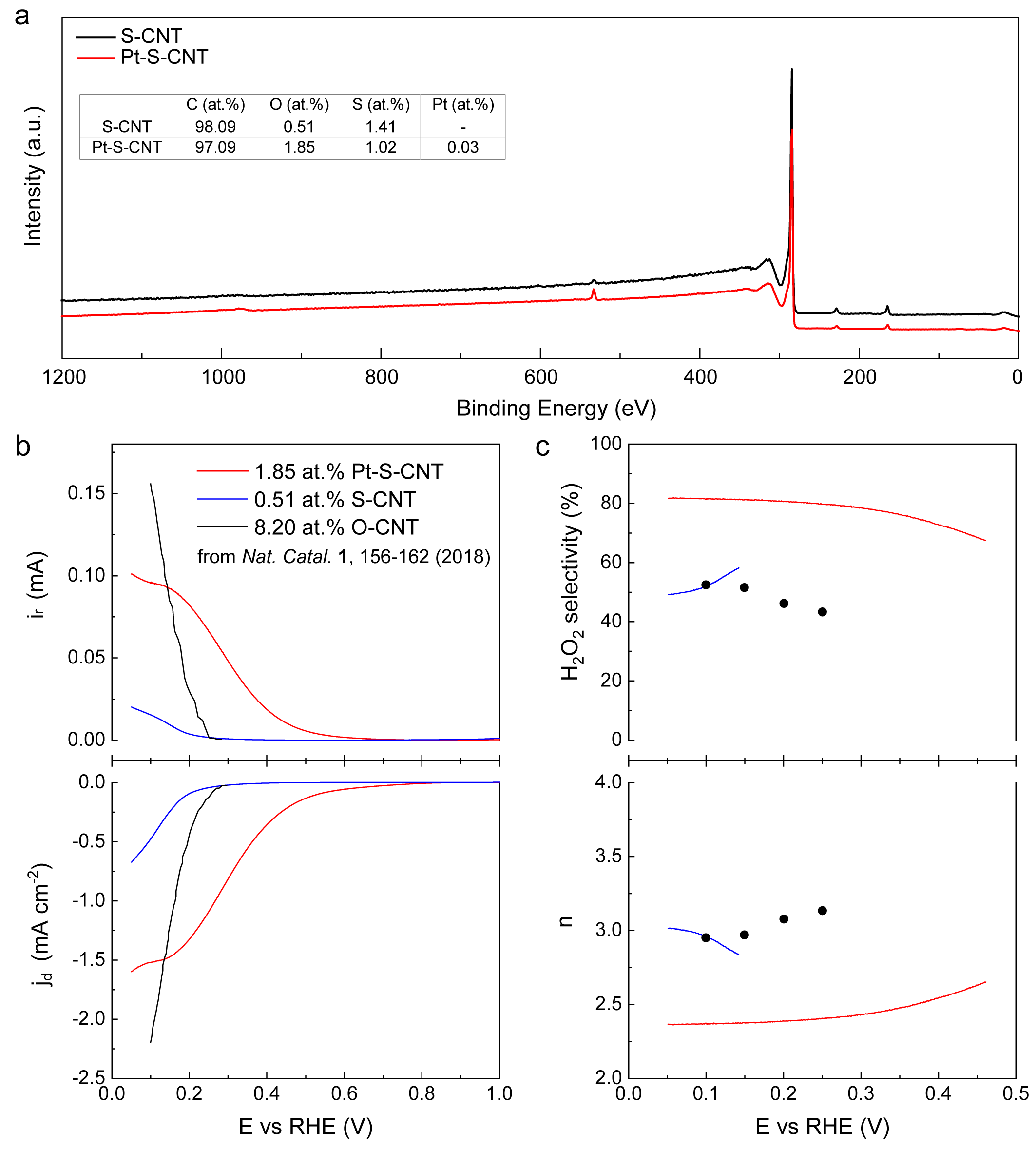


**Supplementary Figure 9.** (**a**) XPS survey spectra for Pt-S-CNT catalysts and S-CNT. The atomic content for each element, together with O-CNT as adapted from Nat. Catal. **1**, 156-162 (2018), are tabulated as insert. (**b**) Linear sweep voltammetry of Pt-S-CNT, S-CNT and O-CNT recorded at 1600 rpm and a scan rate of 5 mV s^−1^, together with the detected H_2_O_2_ currents on the ring electrode (upper panel) at a fixed potential of 1.2 V vs. RHE. The catalyst loading was fixed at 0.1 mg cm^−2^. (**c**) Calculated H_2_O_2_ selectivity and electron transfer number (*n*) during potential sweep. H_2_O_2_ selectivity and *n* were plotted from the onset potential that reached 0.1 mA cm^-2^ H_2_O_2_ partial current density. An earlier onset of ~310 mV plus an enhanced H_2_O_2_ selectivity is noted with the presence of Pt-S moiety, thus highlighting the promotion effect of Pt-S coordination to O_2_-to-H_2_O_2_ conversion.


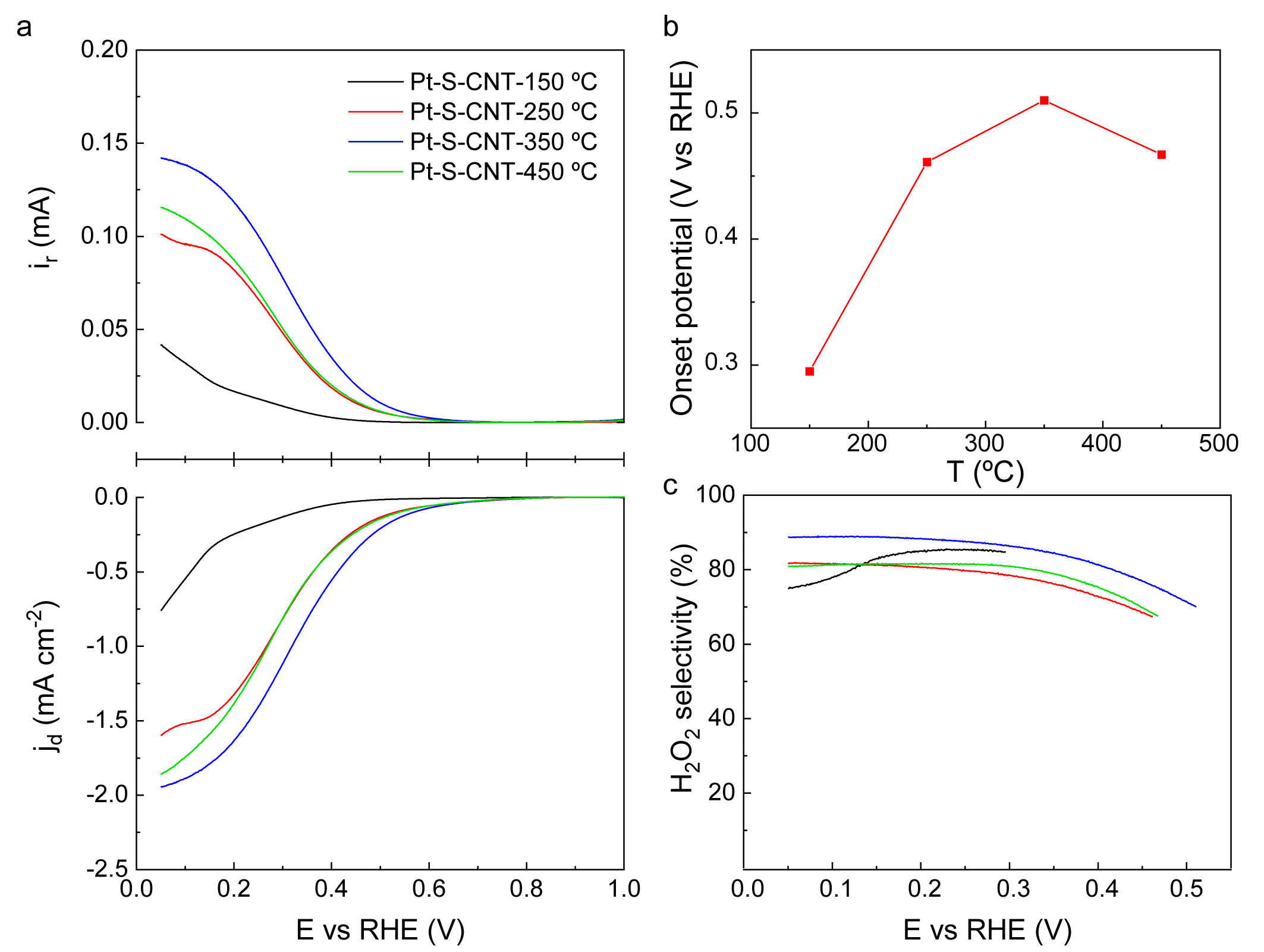


**Supplementary Figure 10**. ORR performance of Pt-S-CNT catalysts prepared at different annealing temperature. (**a**) Linear sweep voltammetry of Pt-S-CNT recorded at 1600 rpm and a scan rate of 5 mV s^−1^, together with the detected H_2_O_2_ currents on the ring electrode (upper panel) at a fixed potential of 1.2 V vs. RHE. The catalyst loading was fixed at 0.1 mg cm^−2^. (**b**) The onset potential of Pt-S-CNT catalysts annealed at different temperature and (**c**) potential-dependence H_2_O_2_ selectivity. The onset potential was defined as that delivers 0.1 mA cm^-2^ H_2_O_2_ partial current density.

**
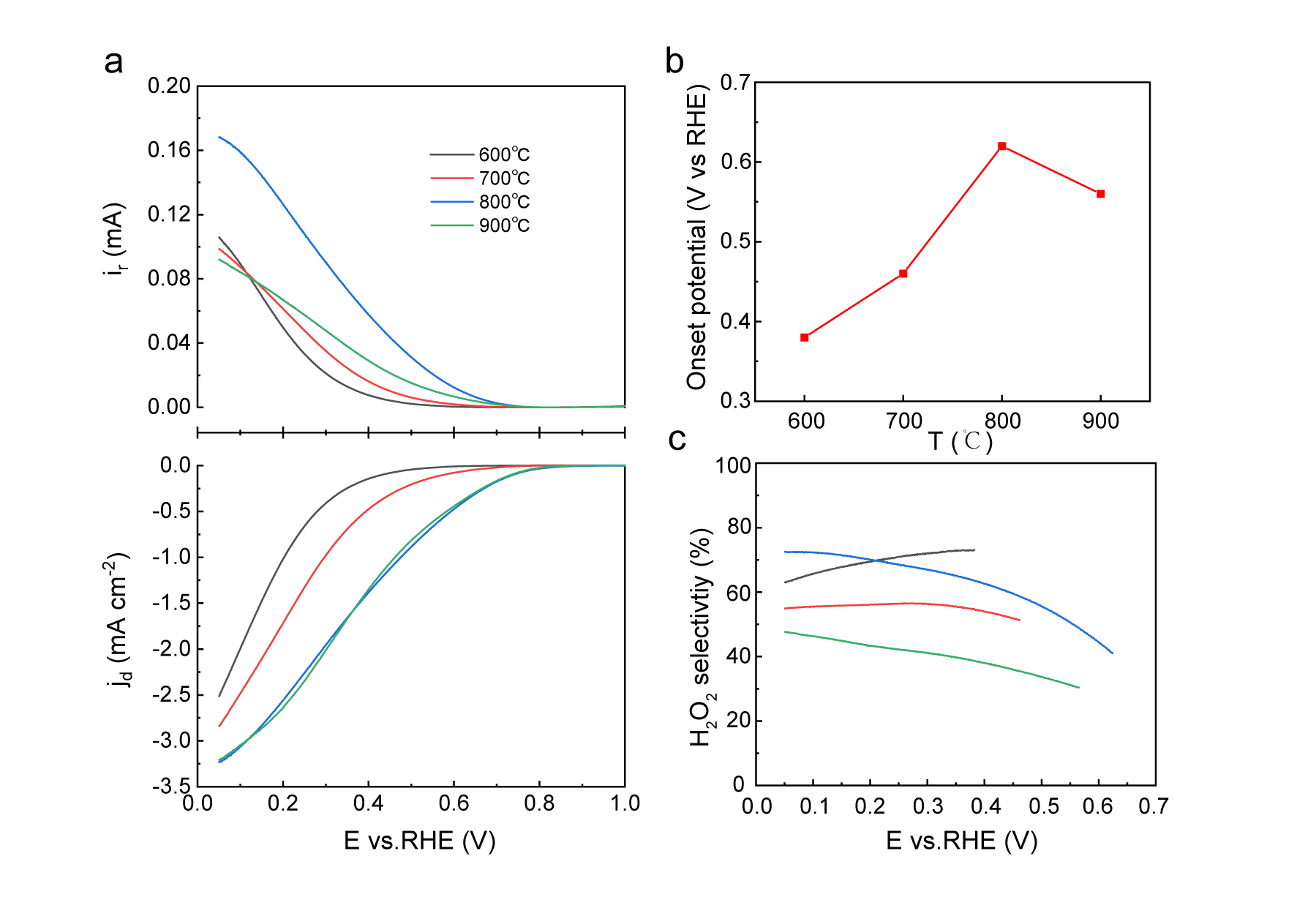
**

**Supplementary Figure 11.** Effect of annealing temperature on the ORR performance of 200-Pt-N-CNT. (**a**) LSVs recorded at 1600 rpm and a scan rate of 5 mV s^−1^, together with the detected H_2_O_2_ currents on the ring electrode (upper panel) at a fixed potential of 1.2 V vs RHE, in 0.1 M O_2_-saturated HClO_4_. (**b**) The onset potential of the Pt-N-CNT catalyst annealed at different temperatures, (**c**) potential-dependence H_2_O_2_ selectivity. The Pt-N-CNT catalyst annealed at 800°C has the most positive onset potential and the largest ring current.


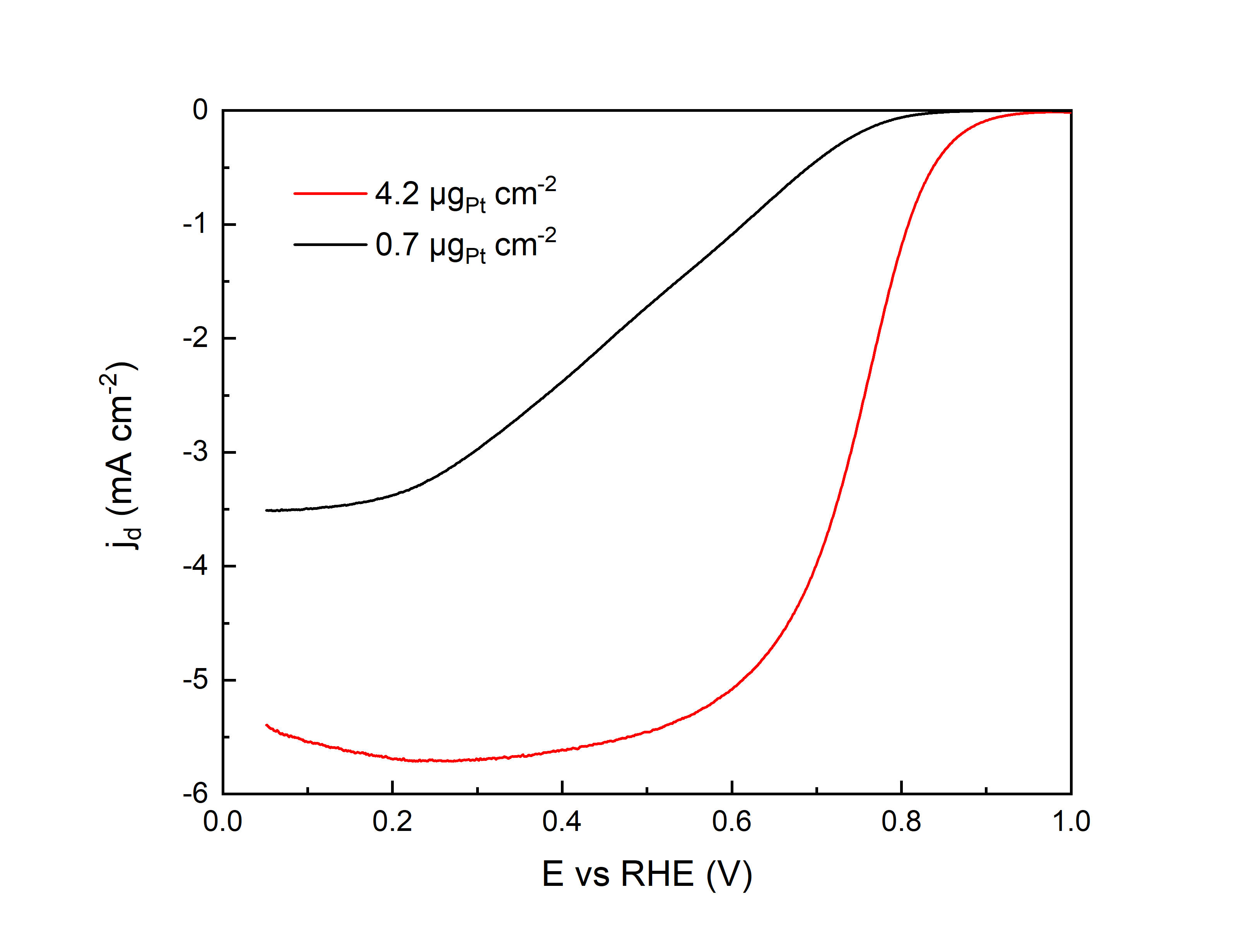


**Supplementary Figure 12.** LSVs of Pt-C-CNT with different Pt loading recorded in 0.1 M O_2_-saturated HClO_4_, at 1600 rpm and a scan rate of 5 mV s^−1^.


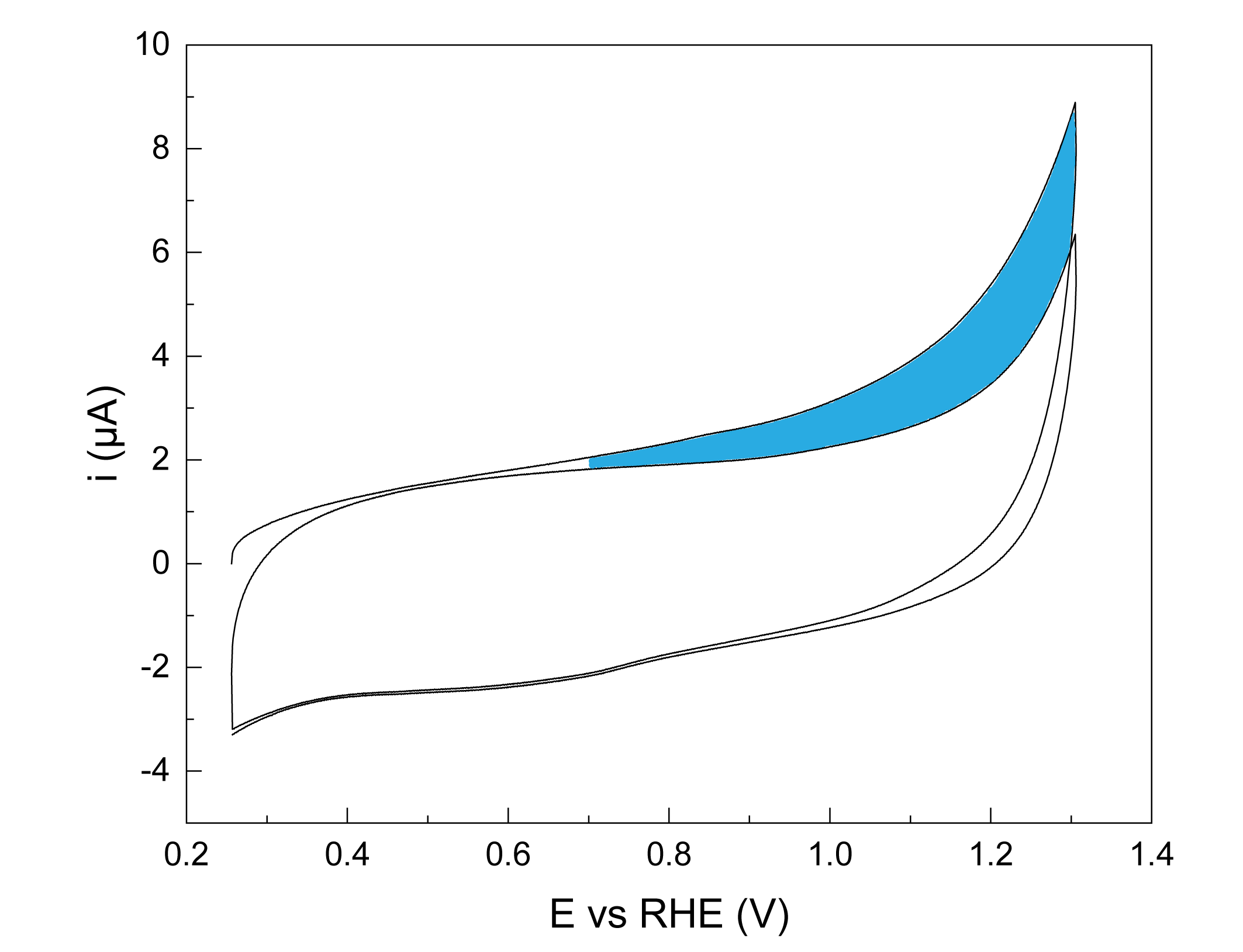


**Supplementary Figure 13.** Representative cyclic voltammograms of electrochemical CO stripping on Pt-S-CNT, scan rate: 5 mV s^-1^. The catalyst loading was fixed at 0.1 mg cm^−2^. The CO_des_ charge integral interval is 0.7-1.3 V vs RHE, as marked in the blue shaded part.


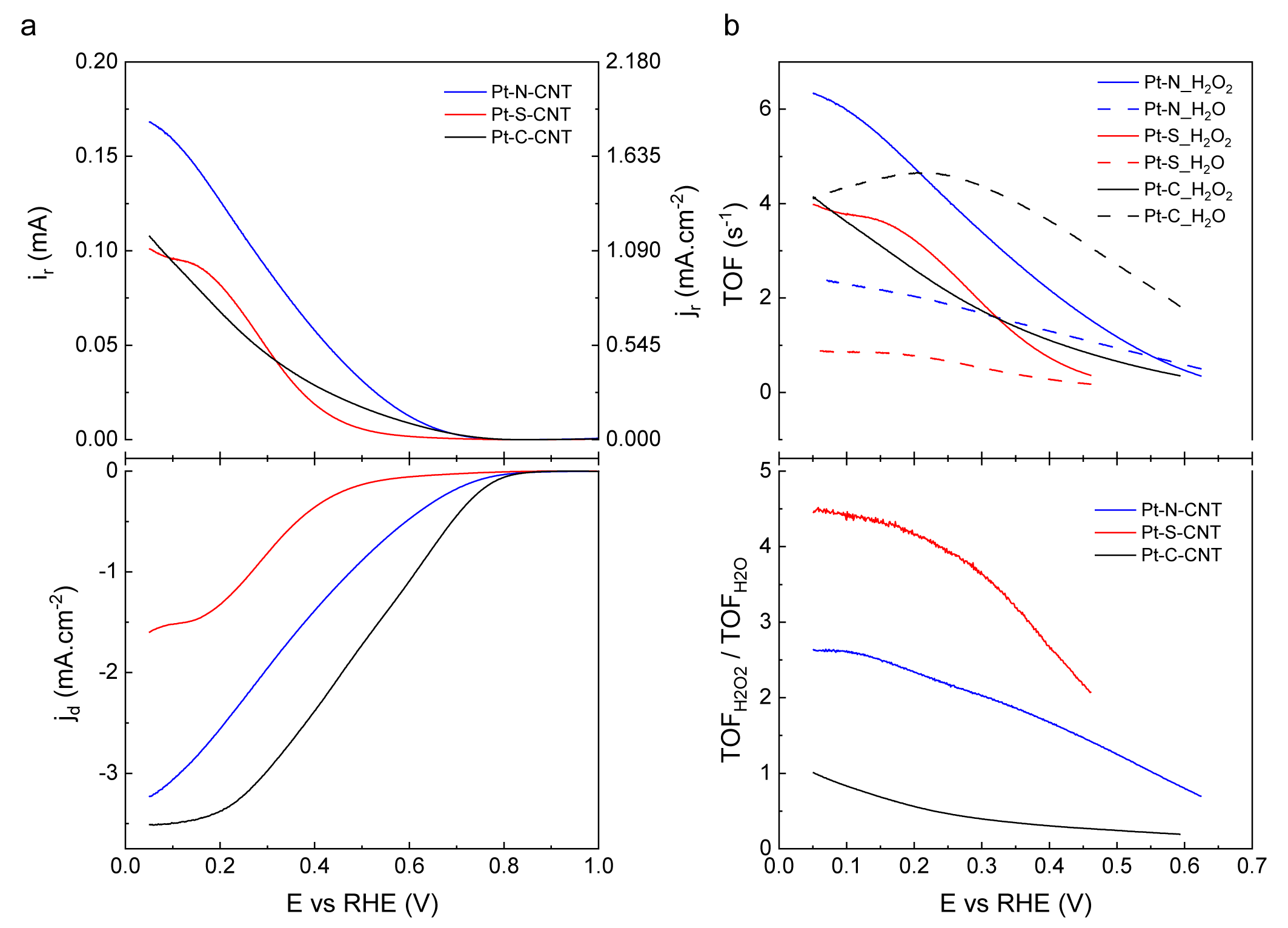


**Supplementary Figure 14.** ORR performance of Pt-X-CNT catalysts cast RRDE in 0.1 M O_2_-saturated HClO_4_. (**a**) Linear sweep voltammograms of Pt-N-CNT (blue), Pt-S-CNT (red) and Pt-C-CNT (black) recorded at 1600 rpm and a scan rate of 5 mV s^−1^, together with the detected H_2_O_2_ currents on the ring electrode (upper panel) at a fixed potential of 1.2 V vs. RHE. The catalyst loading was fixed at 0.1 mg cm^−2^. (**b**) Calculated TOF and TOF_H2O2_/TOF_H2O_ during LSV scan. All data were plotted from the onset potential that reached 0.1 mA cm^-2^ H_2_O_2_ partial current density.


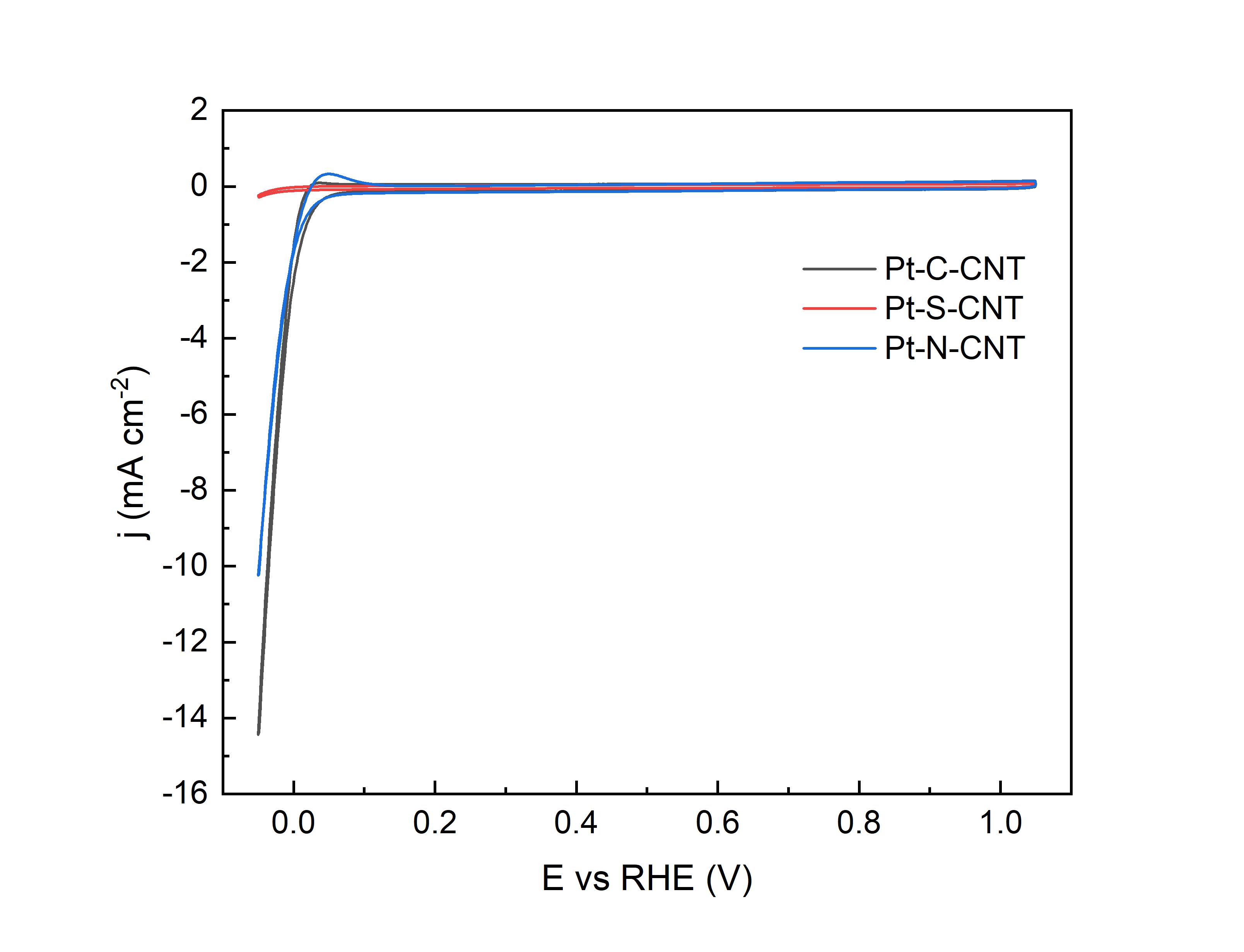


**Supplementary Figure 15.** Cyclic voltammograms of isolated Pt-X-CNT catalysts cast glassy carbon electrode in 0.1 M Ar-saturated HClO_4_. The hydrogen evolution reaction (HER) current in the negative potential sweep follows the trend as Pt-C＞Pt-N＞Pt-S, indicating that changes in the coordination environment of isolated Pt atoms will also alter the HER process. Catalyst loading: 0.1 mg cm^-2^, sweep rate: 50 mV s^−1^.


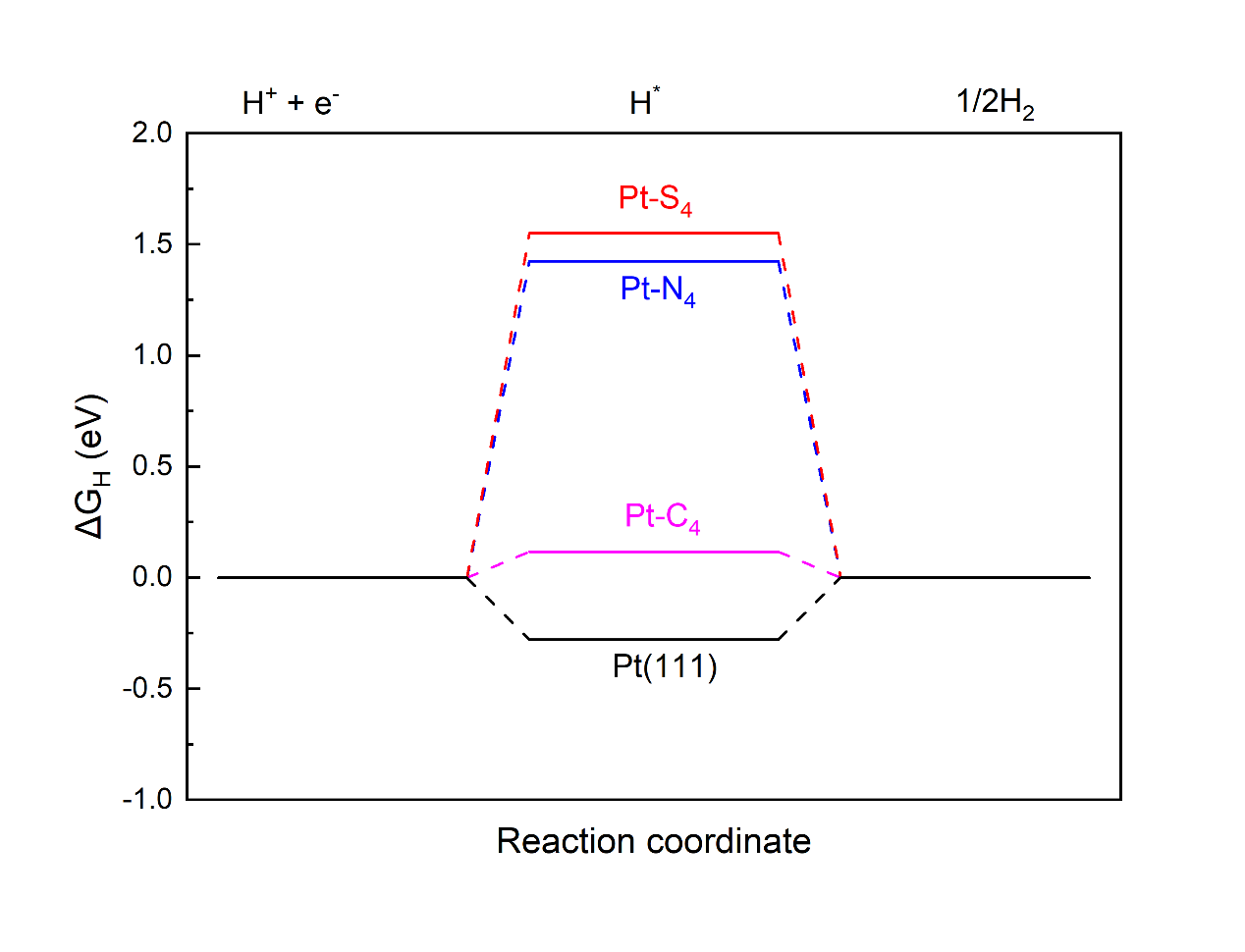


**Supplementary Figure 16.** Free energy diagram of the HER process on representative Pt-coordinated motifs in comparison to Pt(111). Thermodynamically, the HER activity follows the trend as Pt-C>Pt-N>Pt-S, qualitatively explains the above experimental observation shown in **Supplementary Figure 10**.


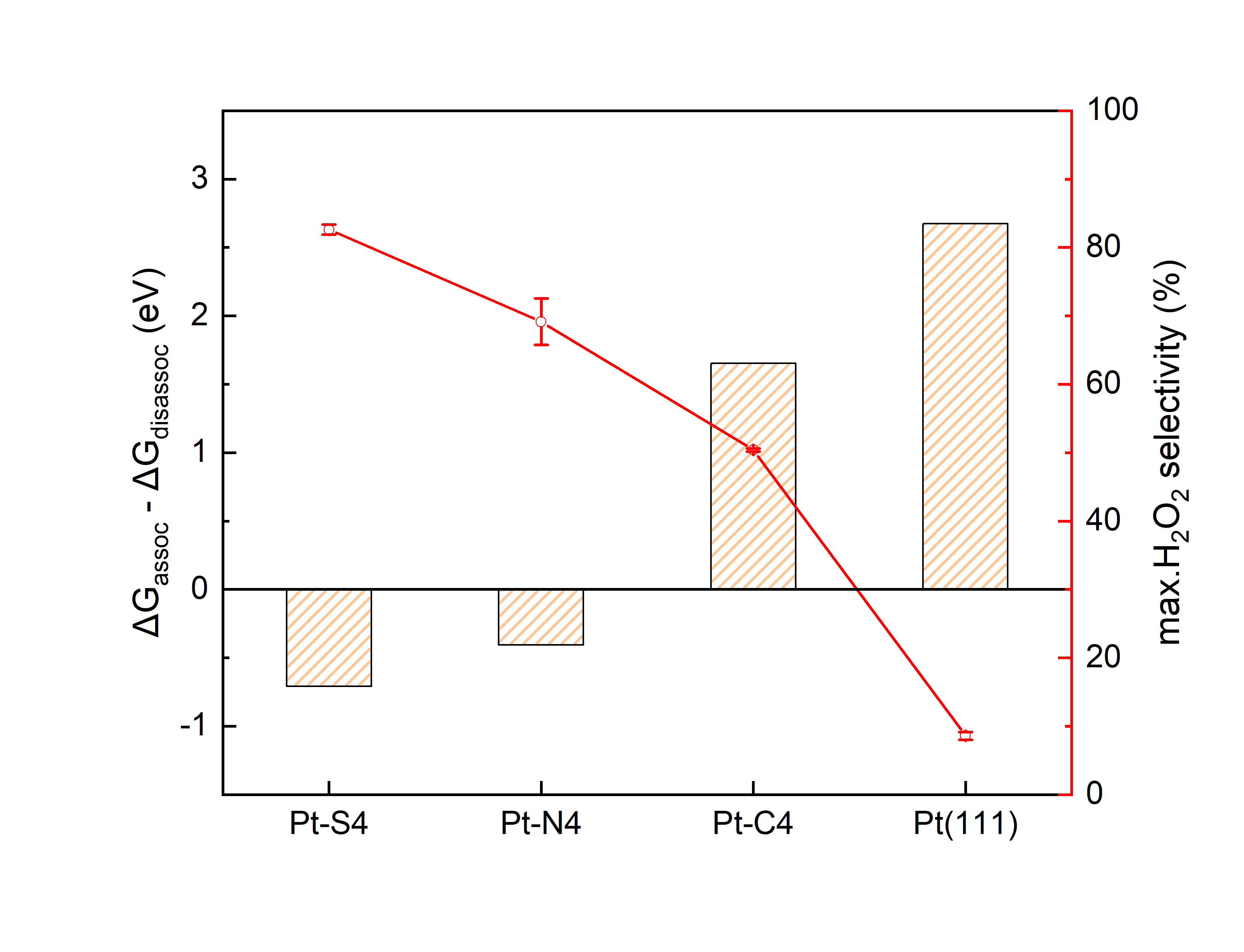


**Supplementary Figure 17.** The relationship between the difference in Gibbs free energy change of OOH* to H_2_O_2_/O* and the experimentally observed H_2_O_2_ selectivity.


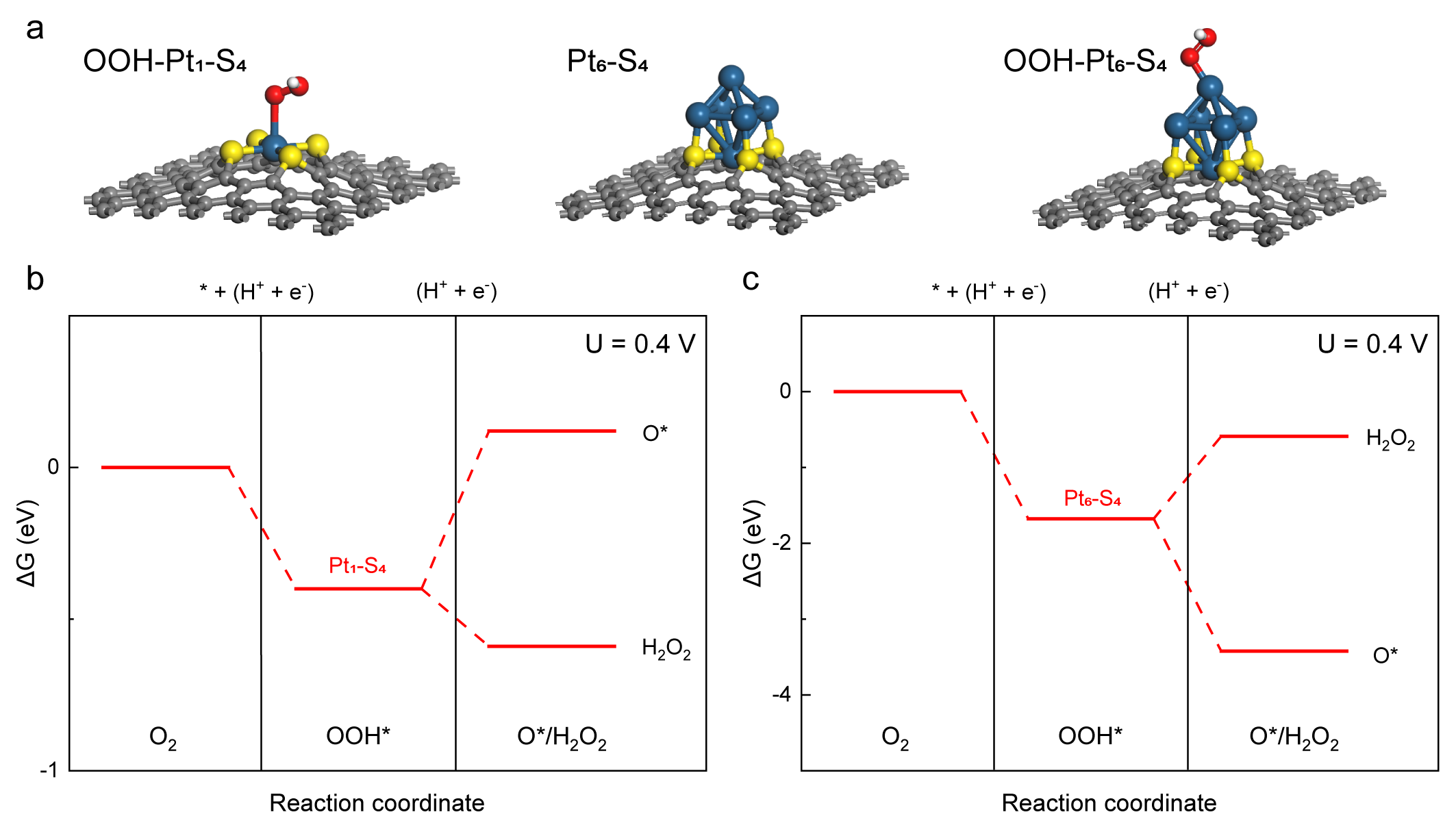


**Supplementary Figure 18**. (**a**) Illustration of Pt_1_-S_4_-C and Pt_6_-S_4_-C motifs, together with the calculated free energy diagrams on (**b**) Pt_1_-S_4_ and (**c**) Pt_6_-S_4_ moieties for ORR process. The energetic difference between 2e^-^ and 4e^-^ ORR pathways (ΔG_assoc_‒ΔG_dissoc_) is ca. -0.71 eV for the former and +2.83 eV for the latter, which means an energetic favorable O_2_-to-H_2_O_2_ conversion on Pt_1_-S_4_ moieties but a favorable O_2_-to-H_2_O conversion on Pt_6_-S_4_ moieties.


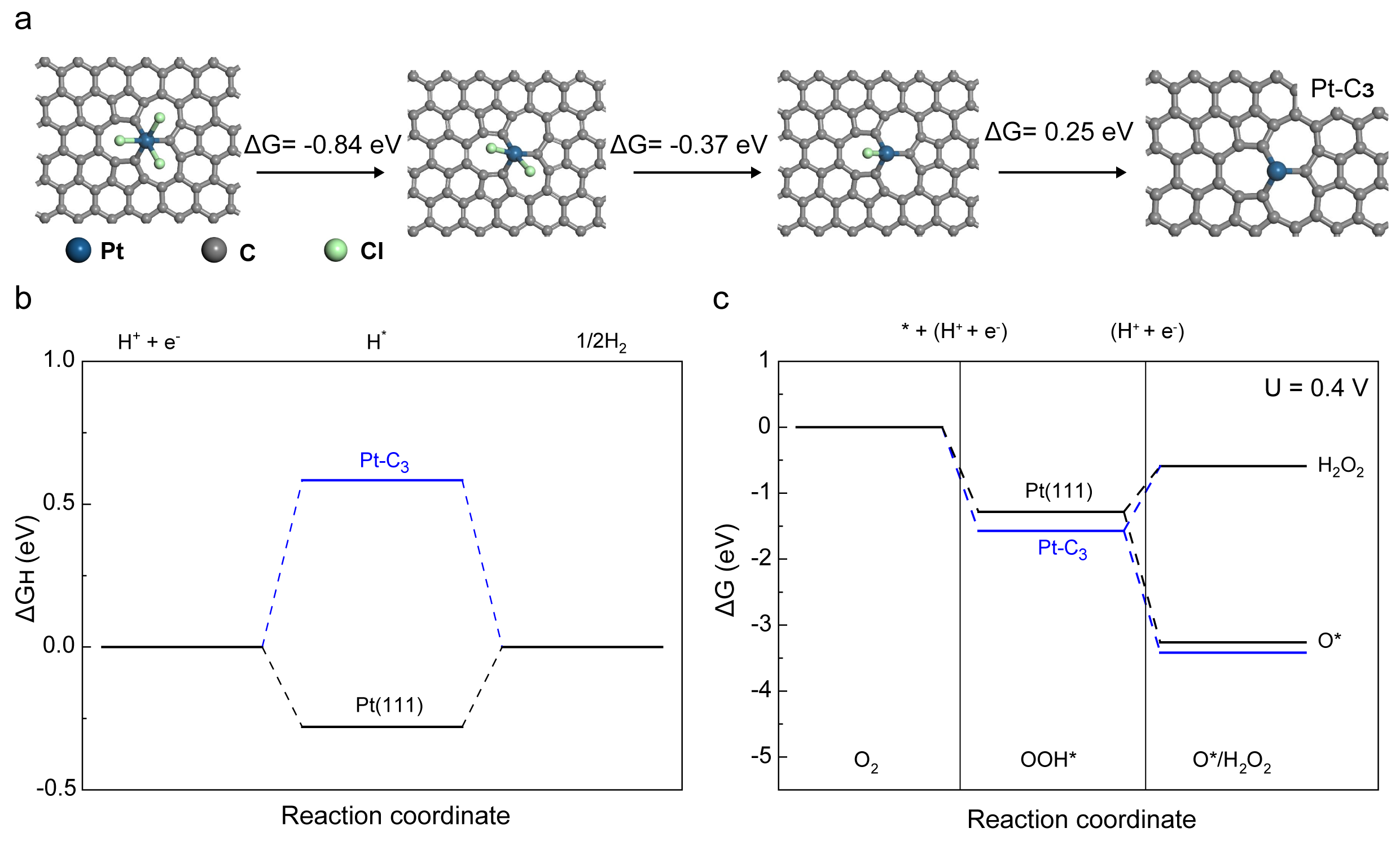


**Supplementary Figure 19.** Theoretical simulations on the Pt-C-Cl moiety (Δ*E_f_* = -3.099 eV) catalyzed HER and ORR. (a) Schematic of the desorption of Cl on the Pt-C_3_-Cl_3_ as suggested in the EXAFS fitting of **Supplementary Fig. 3**. Co-adsorbed chloride species tends to desorb from the central Pt atom during potential cycling. (b) Free energy diagrams for HER and (**c**) ORR process on the representative Pt-C_3_ motif in comparison to Pt(111). Similar to the simulated Pt-C_4_ moiety in **Fig. 3**, the O_2_-to-H_2_O pathway is more energetic favorable on these Pt-C motifs.


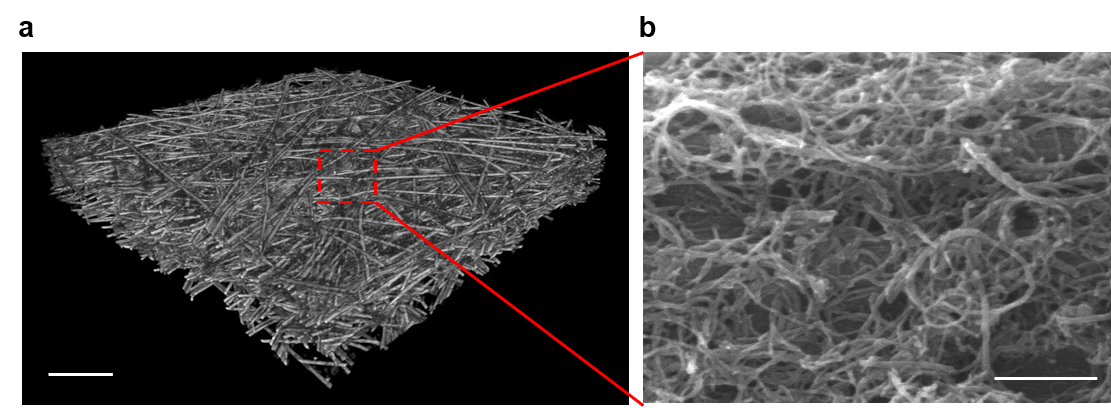


**Supplementary Figure 20.** (**a**) MicroCT 3D tomographic model and (**b**) SEM image of Pt-S-CNT cast CFP. Scale bar is 200 µm in the left panel and 200 nm in the right panel.


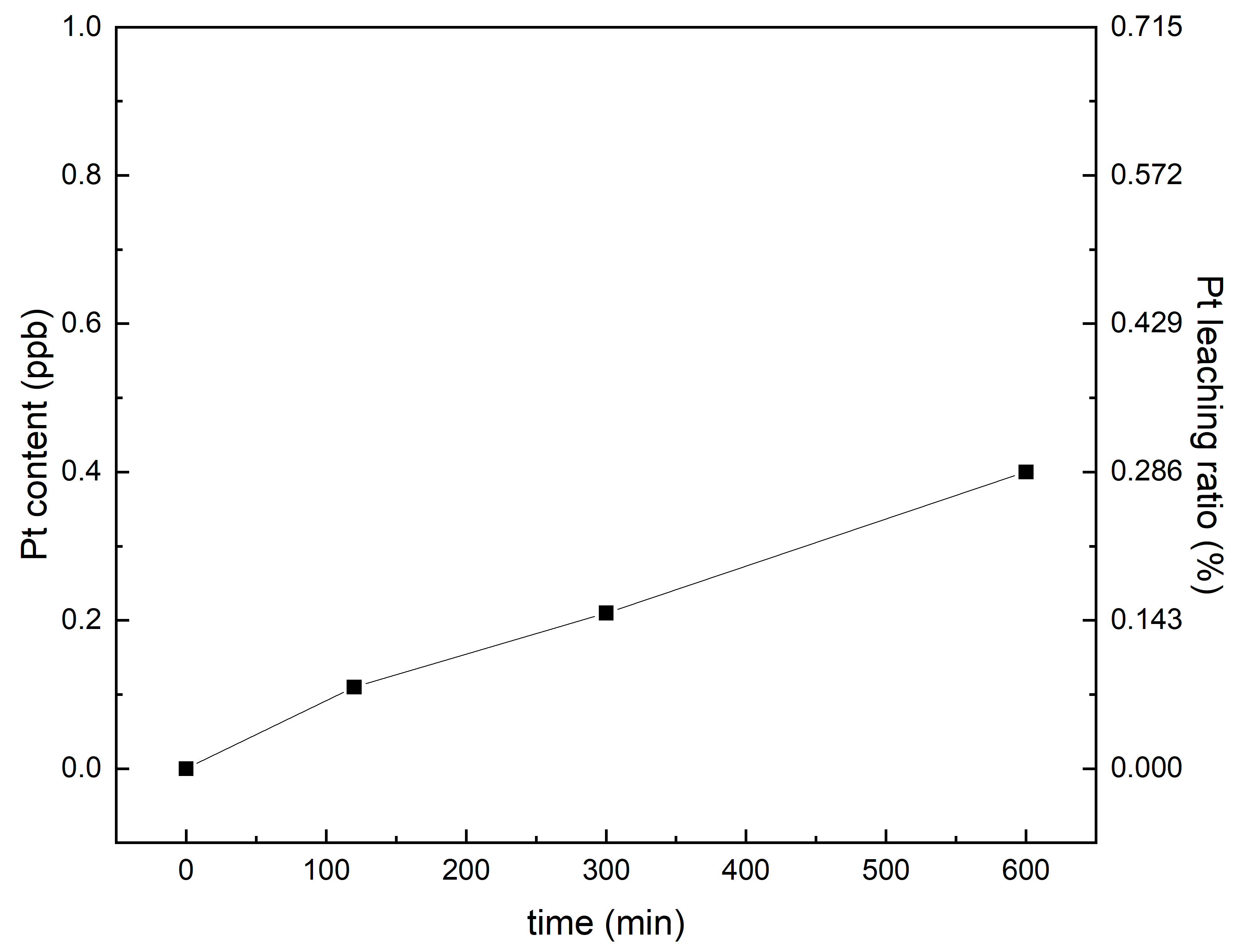


**Supplementary Figure 21**. Time-evolved Pt leaching during the long-term electrolysis as shown in Fig. 4d. After 10 hours’ continuous electrolysis, 0.40 ppb Pt was leached from Pt-S-CNT catalyst, corresponding to 0.29 at.% molar ratio. For the ICP-MS measurements, 2 mL of electrolyte was picked up at each time slot from repeated long-term ORR stability test over Pt-S-CNT (Fig. 4d in Manuscript). If all the Pt was leached out from catalyst, the Pt concentration in electrolyte should be ~140 ppb.


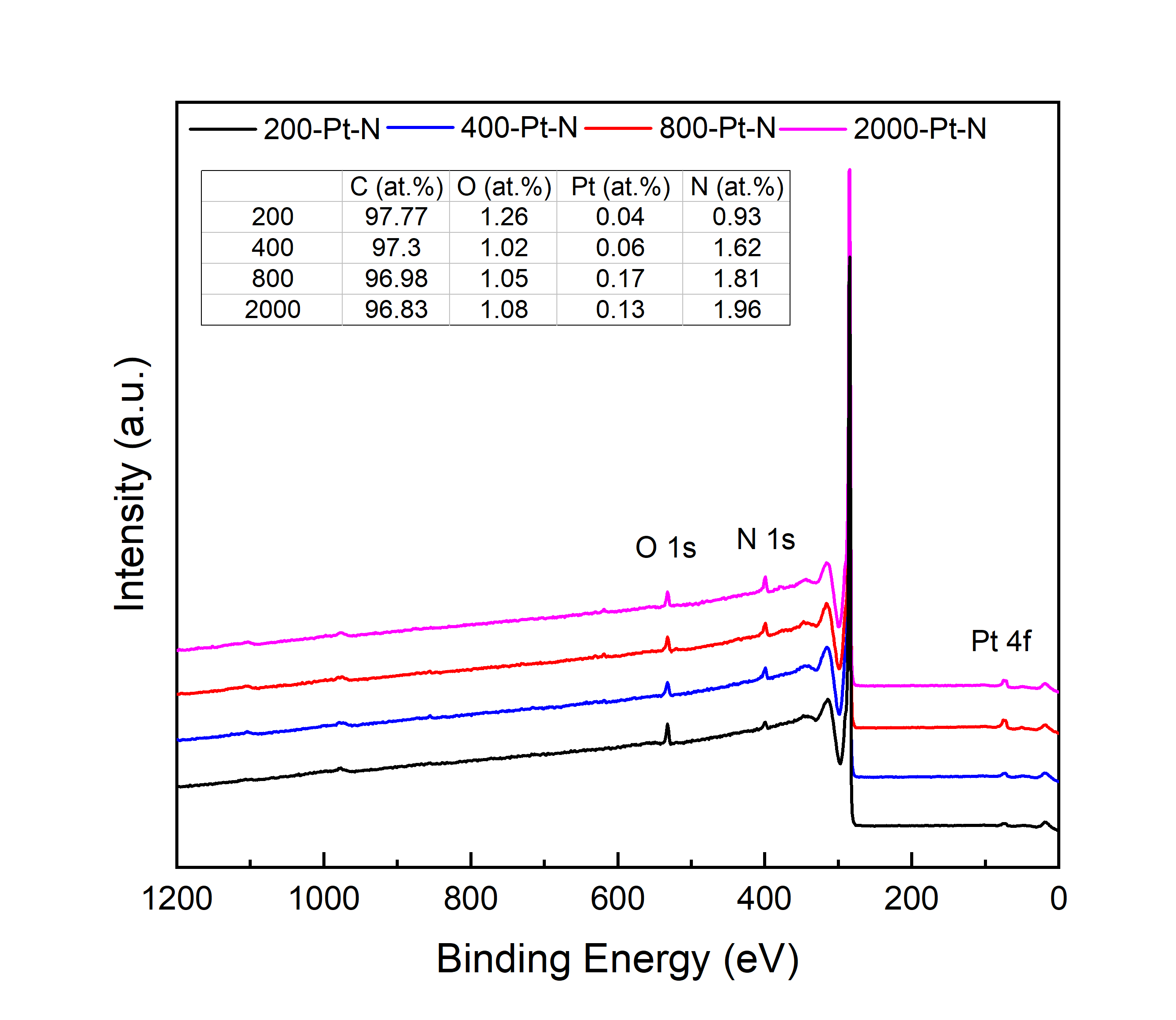


**Supplementary Figure 22.** XPS characterizations of Pt-N-CNT catalysts with different Pt-loading. For all the tested samples, only C, O, N and Pt elements were detected, and their atomic content are tabulated as insert. The detected Pt atomic content increases linearly with increasing Pt loading in catalysts for 200-, 400- and 800-Pt-N-CNT, while even higher Pt-loading in 2000-Pt-N-CNT leads to the formation of Pt agglomerate and the (sub)surface Pt content as detected by XPS virtually decreases.


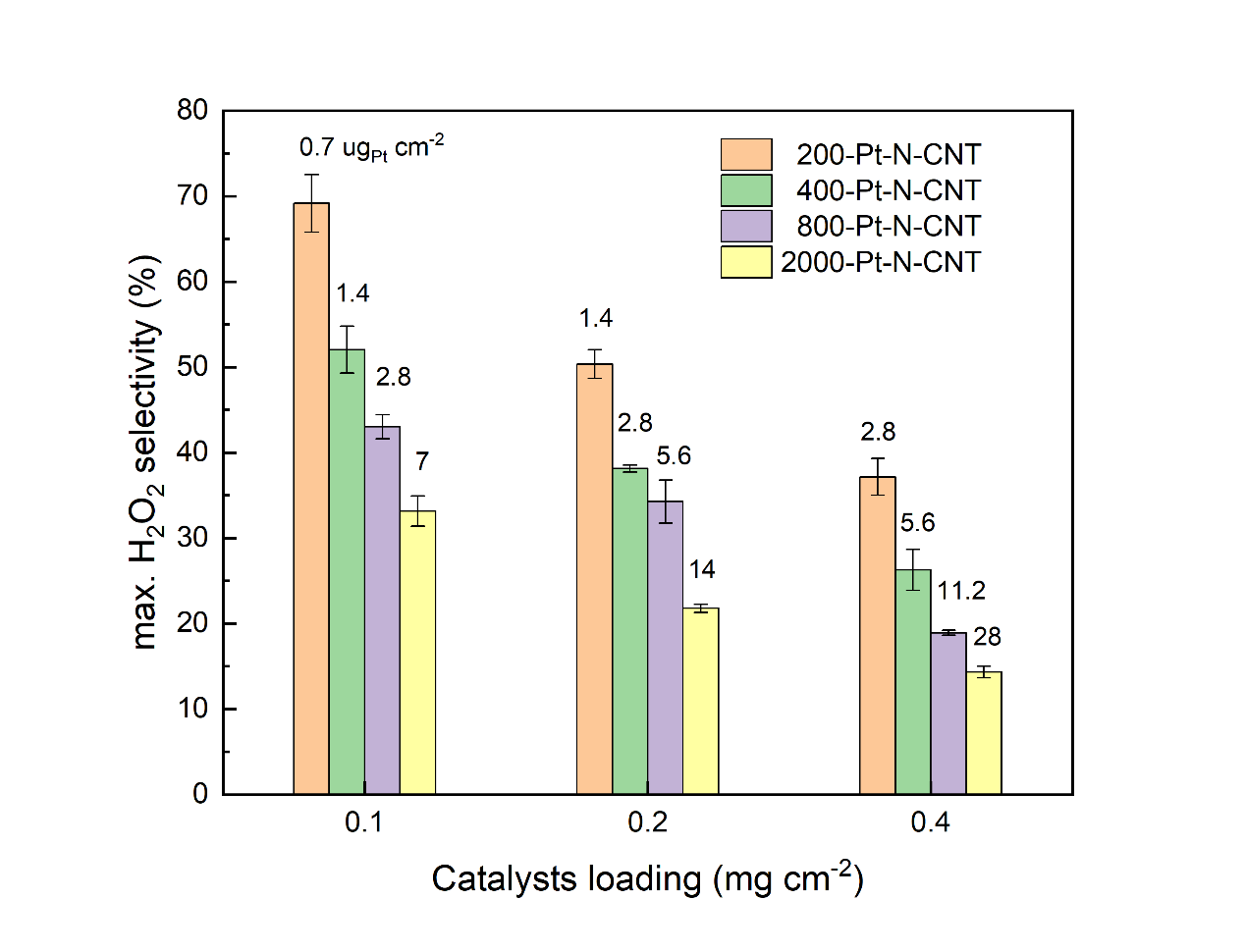


**Supplementary Figure 23.** The dependence of maximum H_2_O_2_ selectivity as a function of either catalyst loading (x axis) or Pt sites density (colored bars). The increases in either Pt sites density or catalyst loading will tune down the maximum H_2_O_2_ selectivity.


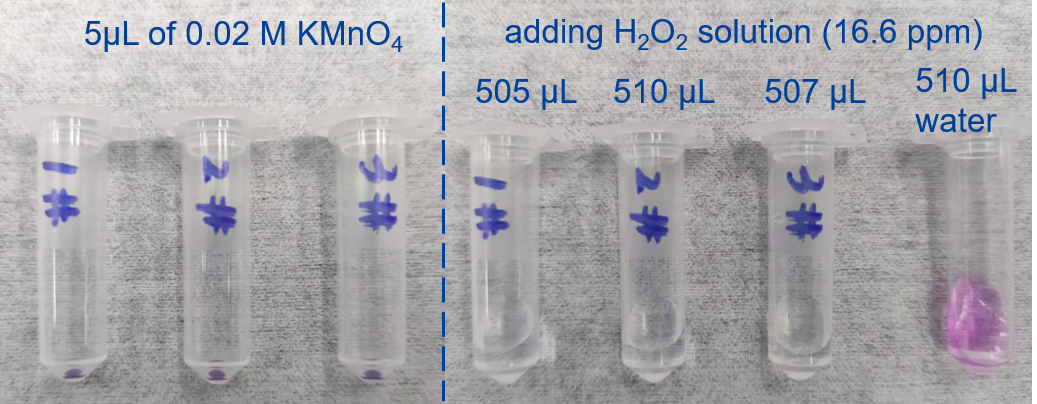


**Supplementary Figure 24.** H_2_O_2_ detection limit for KMnO_4_ titration method. The left side is the KMnO_4_ solution, and the right side is the solution after adding H_2_O_2_ or water.

To determine the detection limit of KMnO_4_ titration, 1 μL of 30 wt.% H_2_O_2_ (GR, Sinopharm) was firstly diluted in 20 mL of Millipore water (ca. 16.6 ppm H_2_O_2_), then being added dropwise to a test tube containing 5 μL of 0.02 M KMnO_4_ until the solution becomes colorless (Tube 1#, 2# and 3#). We repeated it three times, and the amount of H_2_O_2_ solution consumed was 505 μL, 510 μL and 507 μL, respectively, which was close to the theoretical value 510.5 μL that calculated from the reaction equation: 2MnO_­4_^-^ + 5H_2_O_2_ +6H^+^ → 6Mn_2_^+^ + 5O_2_ + 8H_2_O. The solution in the test tube of a control group (rightmost one) was still purple with 510 μL of water added, indicating that dilution does not make KMnO_4_ colorless. To titrate the unknown H_2_O_2_ content in the bulk electrolysis solution, 1 mL of electrolyte was collected instead of the above standard 16.6-ppm H_2_O_2_ solution, therefore, the detection limit of this KMnO_4_ titration can be calculated as 16.6×510.5/1000=8.5 ppm, which is lower than the first data point we measured in Figure 4e (ca. 71.7 ± 2.5 ppm from two independent measurements).


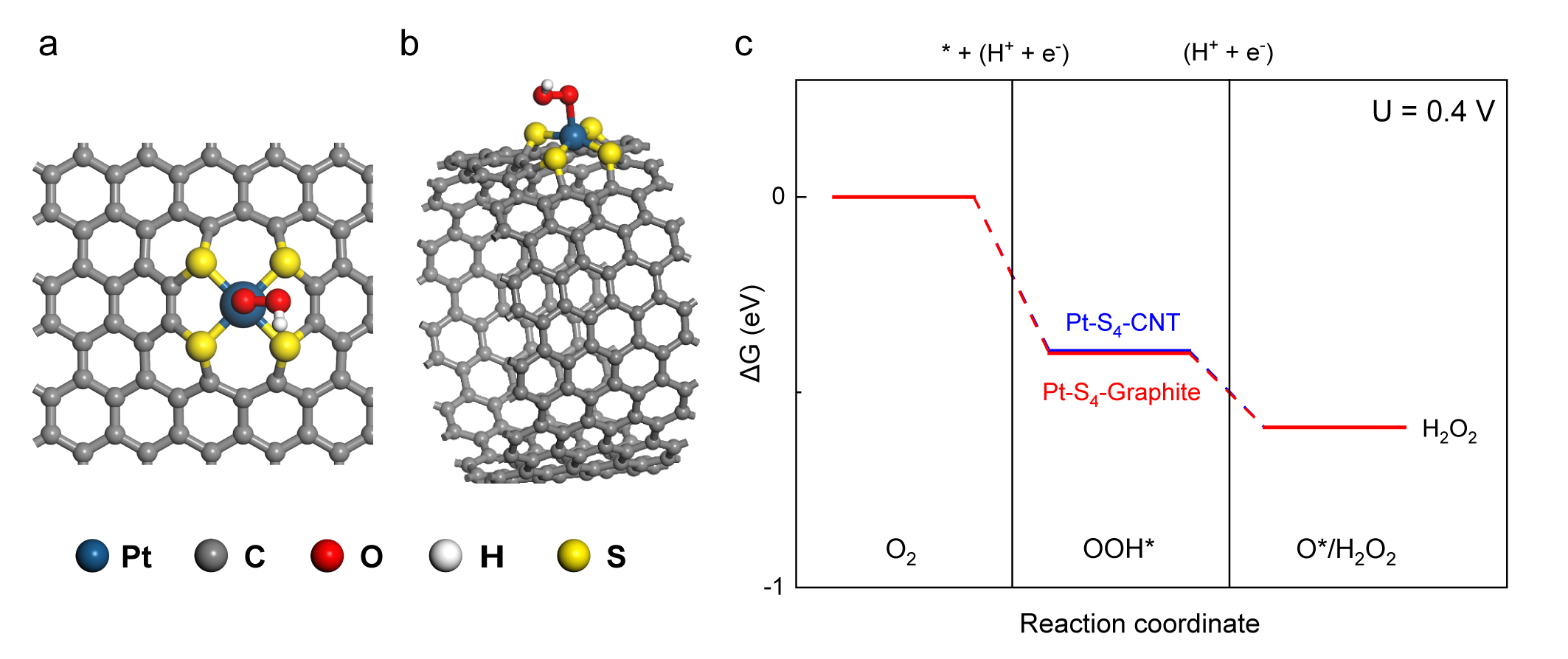


**Supplementary Figure 25**. Curvature effect evaluation on O_2_-to-H_2_O_2_ conversion. (**a**) Illustration of Pt-S_4_ motif anchored on either monolayer graphite or (**b**) curved carbon nanotube substrate. (**c**) Calculated free energy diagrams on Pt-S-Graphite and Pt-S-CNT moieties. Nearly identical *OOH adsorption strength is revealed, suggestive a minor curvature effect on H_2_O_2_ generation.

**Supplementary Table 1.** Electrocatalytic O_2_-to-H_2_O_2_ conversion performance summary.

| Catalyst | j_d_ *^a^* (mA/cm^2^) | max. H_2_O_2_  (%) | E_onset_ vs. RHE *^b^* (V) | Electrolyte | Ref. |
| --- | --- | --- | --- | --- | --- |
| Pt-S-CNT | -1.6 | 81.2 | 0.46 | 0.1 M HClO_4_ | This work |
| Pt-Hg | -3.9 | 96 | 0.52 | 0.1 M HClO_4_ | *Nat. Mater.* **2013,** *12*, 1137-1143^4^ |
| Pt\HSC | -1.6 | 95 | 0.5 | 0.1 M HClO_4_ | *Nat. Commun.* **2016,** *7*, 10922^5^ |
| Pt/TiN | -2.5 | 65 | 0.45 | 0.1 M HClO_4_ | *Angew. Chem. Int. Ed.* **2016,** *55,* 2058-2062^6^ |
| Pt/TiC | -1.63 | 78 | 0.4 | 0.1 M HClO_4_ | *ACS Catal.* **2017,** *7,* 1301-1307^7^ |
| Pt_1_-CuS_x_ | -2.6 | 96 | 0.65 | 0.1 M HClO_4_ | *Chem* **2019,** *5,* 2099-2110^8^ |
| Pt-AuCu | -2.5 | 91.8 | 0.42 | 0.1 M HClO_4_ | *ACS Appl. Energy Mater.* **2019,** *2,* 7722-7727^9^ |
| Pt/CNT_IL_SiO_2_ | -2.0 | 75 | 0.45 | 0.1 M HClO_4_ | *Acs Nano* **2020,** *14,* 1990-2001^10^ |
| Pd-Hg/C | -0.6 | 95 | 0.6 | 0.1 M HClO_4_ | *Nano Lett.* **2014,** *14*, 1603-1608^11^ |
| Au_1‐x_Pd_x_/C | -1.4 | 90 | 0.5 | 0.1 M HClO_4_ | *J. Am. Chem. Soc.* **2011,** *13*3, 19432-19441^12^ |
| CMK-3 | -1.4 | 83 | 0.3 | 0.5 M H_2_SO_4_ | *ACS Catal.* **2018,** *8*, 2844-2856^13^ |
| Co-NC | -3.2 | 90 | 0.6 | 0.1 M HClO_4_ | *Chem* **2020,** *6*, 658-674^14^ |
| CoNOC | -3.0 | 95 | 0.57 | 0.1 M HClO_4_ | J. Am. Chem. Soc. **2021,** *143,* 7819-7827^15^ |
| meso-BMP | -3.5 | 20 | 0.4 | 0.1 M HClO_4_ | *Journal of Energy Chemistry* **2016,** *25*, 251-257^16^ |
| O-CNTs | -2.3 | 52.5 | 0.25 | 0.1 M HClO_4_ | *Nat. Catal.* **2018,** *1*, 156-162^17^ |

*^a^* diffusion-limited current density; *^b^* onset potential is defined as the potential where 0.1 mA cm^-2^ H_2_O_2_ partial current density is achieved

**Supplementary Table 2.** Detailed parameters for the core-level XPS spectra fitting.

| Sample | XPS Peak | | | Position (eV) | FWHM (eV) | Area |
| --- | --- | --- | --- | --- | --- | --- |
| 200-Pt-C-CNT | Pt 4f_7/2_ | | | 72.0 | 1.80 | 105.0 |
|  | Pt 4f_5/2_ | | | 75.3 | 1.80 | 96.0 |
| 200-Pt-S-CNT | Pt 4f_7/2_ | | | 72.5 | 2.00 | 168.5 |
|  | Pt 4f_7/2_ | | | 75.8 | 2.00 | 126.4 |
|  | C-S-C | | S 2p_3/2_ | 164.2 | 0.98 | 547.3 |
|  |  |  | S 2p_1/2_ | 165.4 | 0.98 | 273.6 |
|  | -SO_x_ | | S 2p_3/2_ | 165.9 | 0.98 | 58.3 |
|  |  |  | S 2p_1/2_ | 167.0 | 0.98 | 29.2 |
| 200-Pt-N-CNT | Pt 4f_7/2_ | | | 72.4 | 2.10 | 138.0 |
|  | Pt 4f_5/2_ | | | 75.7 | 2.10 | 130.0 |
|  | N 1s | pyridine-N | | 398.5 | 1.28 | 158.3 |
|  |  | pyrrole-N | | 400.4 | 1.28 | 227.4 |
|  |  | graphitic-N | | 401.6 | 1.28 | 110.3 |
|  |  | oxidized-N | | 403.1 | 1.28 | 71.5 |
|  |  | Pt-N | | 399.2 | 0.98 | 105.0 |
| 400-Pt-N-CNT | Pt 4f_7/2_ | | | 72.4 | 2.18 | 302.2 |
|  | Pt 4f_5/2_ | | | 75.8 | 2.18 | 260.0 |
| 800-Pt-N-CNT | Pt 4f_7/2_ | | | 72.4 | 2.25 | 782.8 |
|  | Pt 4f_5/2_ | | | 75.8 | 2.25 | 672.0 |
| 2000-Pt-N-CNT | Pt^0^ | | Pt 4f_7/2_ | 71.5 | 0.87 | 139.6 |
|  |  |  | Pt 4f_5/2_ | 74.8 | 0.87 | 115.1 |
|  | Pt^δ+^ | | Pt 4f_7/2_ | 72.3 | 2.01 | 520.3 |
|  |  |  | Pt 4f_5/2_ | 75.7 | 2.01 | 425.7 |

**Supplementary Table 3**. The amount of Pt measured by XPS and CO stripping.

| sample | Theoretical loading (wt.%) | measured by XPS (wt.%) | measured by CO stripping (wt.%) |
| --- | --- | --- | --- |
| 200-Pt-S-CNT | 0.70 | 0.50 | 0.28 |
| 200-Pt-C-CNT | 0.70 | 0.49 | 0.29 |
| 200-Pt-N-CNT_0.1mg cm^-2^ | 0.70 | 0.66 | 0.29 |
| 200-Pt-N-CNT_0.2mg cm^-2^ | 1.40 | - | 0.60 |
| 200-Pt-N-CNT_0.4mg cm^-2^ | 2.80 | - | 1.26 |
| 400-Pt-N-CNT | 1.40 | 0.96 | 0.36 |
| 800-Pt-N-CNT | 2.80 | 2.68 | 0.47 |
| 2000-Pt-N-CNT | 7.00 | 2.06 | 0.51 |

**Supplementary references**

1 Liu, J. *et al.* High performance platinum single atom electrocatalyst for oxygen reduction reaction. *Nat. Commun.* **8**, 15938 (2017).

2 Wang, X. X. *et al.* Nitrogen-coordinated single cobalt atom catalysts for oxygen reduction in proton exchange membrane fuel cells. *Adv. Mater.* **30**, 1706758 (2018).

3 Hoque, M. A. *et al.* Web-like 3d architecture of Pt nanowires and sulfur-doped carbon nanotube with superior electrocatalytic performance. *ACS Sustain. Chem. Eng.* **6**, 93-98 (2018).

4 Siahrostami, S. *et al.* Enabling direct H_2_O_2_ production through rational electrocatalyst design. *Nat. Mater.* **12**, 1137-1143 (2013).

5 Choi, C. H. *et al.* Tuning selectivity of electrochemical reactions by atomically dispersed platinum catalyst. *Nat. Commun.* **7**, 10922 (2016).

6 Yang, S., Kim, J., Tak, Y. J., Soon, A. & Lee, H. Single-atom catalyst of platinum supported on titanium nitride for selective electrochemical reactions. *Angew. Chem. Int. Ed.* **55**, 2058-2062 (2016).

7 Yang, S., Tak, Y. J., Kim, J., Soon, A. & Lee, H. Support effects in single-atom platinum catalysts for electrochemical oxygen reduction. *ACS Catal.* **7**, 1301-1307 (2017).

8 Shen, R. *et al.* High-concentration single atomic Pt sites on hollow cusx for selective O_2_ reduction to H_2_O_2_ in acid solution. *Chem* **5**, 2099-2110 (2019).

9 Shi, Q. *et al.* Highly dispersed platinum atoms on the surface of AuCu metallic aerogels for enabling H_2_O_2_ production. *ACS Appl. Energy Mater.* **2**, 7722-7727 (2019).

10 Kim, J. H. *et al.* A general strategy to atomically dispersed precious metal catalysts for unravelling their catalytic trends for oxygen reduction reaction. *Acs Nano* **14**, 1990-2001 (2020).

11 Verdaguer-Casadevall, A. *et al.* Trends in the electrochemical synthesis of H_2_O_2_: Enhancing activity and selectivity by electrocatalytic site engineering. *Nano Lett.* **14**, 1603-1608 (2014).

12 Jirkovsky, J. S. *et al.* Single atom hot-spots at Au-Pd nanoalloys for electrocatalytic H_2_O_2_ production. *J. Am. Chem. Soc.* **133**, 19432-19441 (2011).

13 Sun, Y. *et al.* Efficient electrochemical hydrogen peroxide production from molecular oxygen on nitrogen-doped mesoporous carbon catalysts. *ACS Catal.* **8**, 2844-2856 (2018).

14 Gao, J. *et al.* Enabling direct H_2_O_2_ production in acidic media through rational design of transition metal single atom catalyst. *Chem* **6**, 658-674 (2020).

15 Tang, C. *et al.* Tailoring acidic oxygen reduction selectivity on single-atom catalysts via modification of first and second coordination spheres. *J. Am. Chem. Soc.* **143**, 7819-7827 (2021).

16 Hasche, F., Oezaslan, M., Strasser, P. & Fellinger, T.-P. Electrocatalytic hydrogen peroxide formation on mesoporous non-metal nitrogen-doped carbon catalyst. *J. Energy Chem.* **25**, 251-257 (2016).

17 Lu, Z. *et al.* High-efficiency oxygen reduction to hydrogen peroxide catalysed by oxidized carbon materials. *Nat. Catal.* **1**, 156-162 (2018).
